# Mixed Valence {Ni^2+^Ni^1+^} Clusters as Models of Acetyl Coenzyme A Synthase Intermediates

**DOI:** 10.1021/jacs.4c06241

**Published:** 2024-07-18

**Authors:** Daniel W. N. Wilson, Benedict C. Thompson, Alberto Collauto, Reagan X. Hooper, Caroline E. Knapp, Maxie M. Roessler, Rebecca A. Musgrave

**Affiliations:** † Department of Chemistry, King’s College London, 7 Trinity Street, London SE1 1DB, U.K.; ‡ Department of Chemistry, University College London, 20 Gordon Street, London WC1H 0AJ, U.K.; § Department of Chemistry and Centre for Pulse EPR Spectroscopy, Imperial College London, 82 Wood Lane, London W12 0BZ, U.K.; ∥ Stanford PULSE Institute, SLAC National Accelerator Laboratory, Menlo Park, California 94025, United States

## Abstract

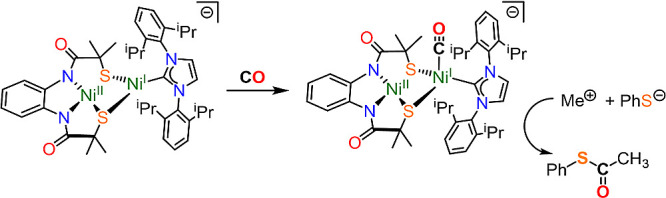

Acetyl coenzyme A synthase (ACS) catalyzes the formation and deconstruction of the key biological metabolite, acetyl coenzyme A (acetyl-CoA). The active site of ACS features a {NiNi} cluster bridged to a [Fe_4_S_4_]^*n*+^ cubane known as the A-cluster. The mechanism by which the A-cluster functions is debated, with few model complexes able to replicate the oxidation states, coordination features, or reactivity proposed in the catalytic cycle. In this work, we isolate the first bimetallic models of two hypothesized intermediates on the paramagnetic pathway of the ACS function. The heteroligated {Ni^2+^Ni^1+^} cluster, [K(12-crown-4)_2_][**1**], effectively replicates the coordination number and oxidation state of the proposed “A_red_” state of the A-cluster. Addition of carbon monoxide to [**1**]^−^ allows for isolation of a dinuclear {Ni^2+^Ni^1+^(CO)} complex, [K(12-crown-2)_*n*_][**2**] (*n* = 1–2), which bears similarity to the “A_NiFeC_*”* enzyme intermediate. Structural and electronic properties of each cluster are elucidated by X-ray diffraction, nuclear magnetic resonance, cyclic voltammetry, and UV/vis and electron paramagnetic resonance spectroscopies, which are supplemented by density functional theory (DFT) calculations. Calculations indicate that the pseudo-T-shaped geometry of the three-coordinate nickel in [**1**]^**–**^ is more stable than the Y-conformation by 22 kcal mol^–1^, and that binding of CO to Ni^1+^ is barrierless and exergonic by 6 kcal mol^–1^. UV/vis absorption spectroscopy on [**2**]^−^ in conjunction with time-dependent DFT calculations indicates that the square-planar nickel site is involved in electron transfer to the CO π*-orbital. Further, we demonstrate that [**2**]^−^ promotes thioester synthesis in a reaction analogous to the production of acetyl coenzyme A by ACS.

## Introduction

**Figure 1:**
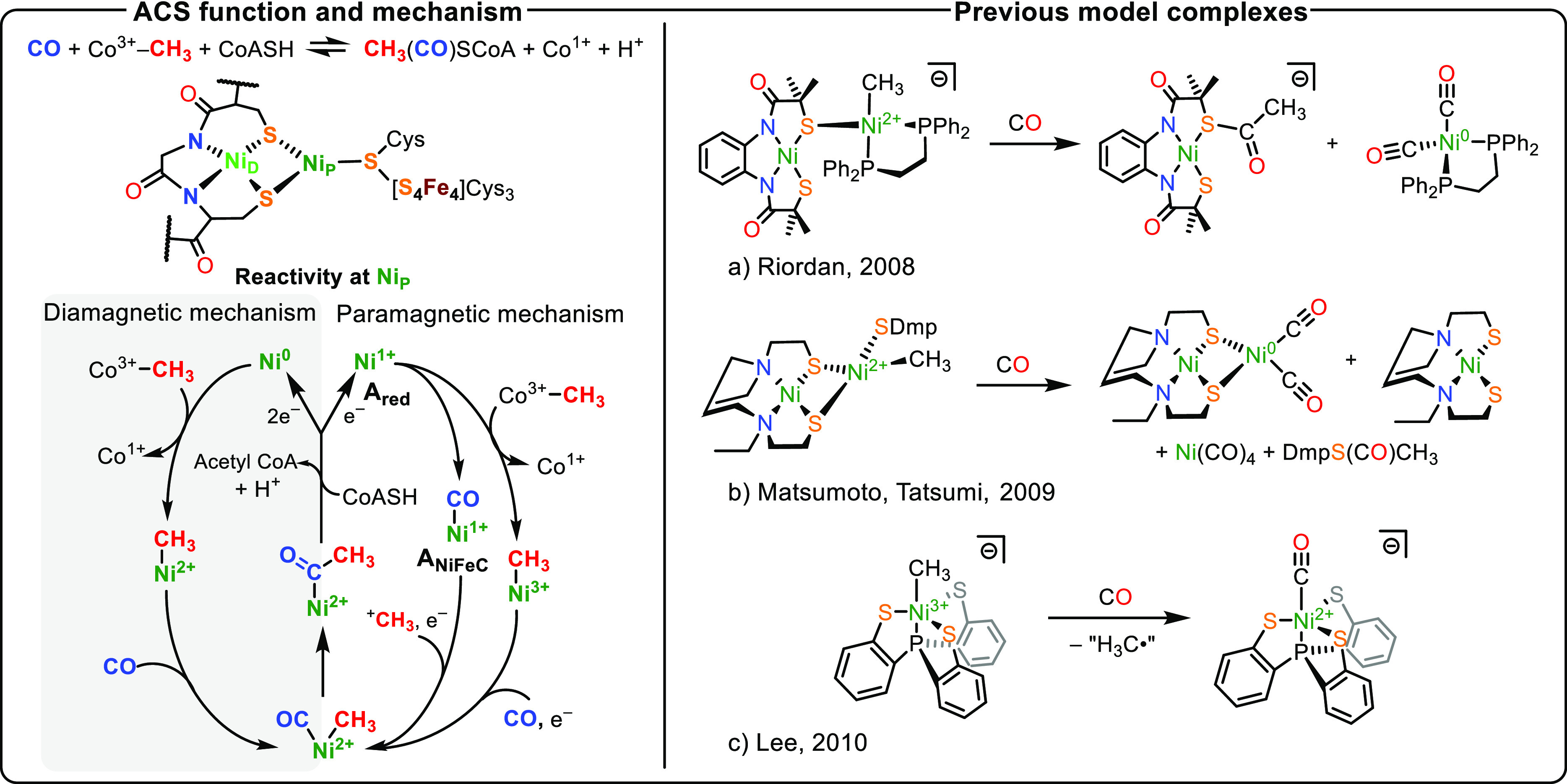
Left (Top): Reversible formation of acetyl coenzyme A (acetyl-CoA) catalyzed by ACS. Middle: The cofactor of acetyl coenzyme A synthase (A-cluster). Bottom: Simplified diamagnetic and paramagnetic mechanisms for the production of acetyl-CoA at the Ni_P_ site of the A-cluster, including the **A**_**red**_ and **A**_**NiFeC**_ intermediates relevant to this work. Right: examples of ACS model complexes and their reactivity toward carbon monoxide.^9−12^

The Wood-Ljundahl pathway (WLP) outlines the conversion of carbon dioxide (CO_2_) into the key biological metabolite acetyl coenzyme A (acetyl-CoA) by a series of bacterial enzymes (Figure S1).^1,2^ The WLP serves as an inspirational example of CO_2_ sequestration: approximately 10^11^ tons of atmospheric CO_2_ are removed by this process every year.^3^ One central transformation to the WLP is the reduction of CO_2_ to carbon monoxide (CO) at the enzyme carbon monoxide dehydrogenase (CODH), and the resulting CO is transferred to the enzyme acetyl coenzyme A synthase (ACS), where it is combined with a CO_2_-derived methyl fragment to form an acetyl group (Figure S1).^4,5^ Finally, this acetyl group is combined with coenzyme A to form acetyl coenzyme A, a thioester used for energy storage and as a source of cellular carbon.^6^ The construction of acetyl-CoA by ACS is performed at a multimetallic cofactor, known as the A-cluster, which features two nickel atoms, termed proximal (Ni_P_) and distal (Ni_D_), which are bridged by two μ_2_-thiolates (Figure 1). The Ni_P_ is also linked to a [Fe_4_S_4_]^*n*+^ cluster by a μ_2_-thiolate, which creates an unusual three-coordinate environment at Ni_P_.^5,7,8^

The production of acetyl-CoA by the A-cluster is thought to involve a series of nickel-based organometallic species in which substrate binding and transformation occur exclusively at the Ni_P_ site.^4,5,13−17^ Multiple divergent mechanisms for ACS activity have been proposed, which can be grouped into the diamagnetic (which features only Ni^0/2+^) and paramagnetic (which features Ni^1+/2+/3+^) mechanisms (Figure 1).^18^ Together, the two pathways invoke Ni-carbonyl, -methyl, and -acyl moieties and place the Ni_P_ site in hypothetical Ni^0^, Ni^1+^, Ni^2+^, and Ni^3+^ oxidation states. Such a wide range of oxidation states is unprecedented in model complexes with ligand environments similar to that of the A-cluster.^4^ Of the many enzyme intermediates proposed in both mechanisms, only the paramagnetic mechanisms’ Ni^1+^–CO adduct (**A**_**NiFeC**_; Figure 1) has been structurally and spectroscopically characterized,^14,16,19^ although its relevance to the function of ACS is a topic of ongoing debate.^20−22^ The lack of further detectable intermediates from the paramagnetic pathway may be due to challenges in studying the enzyme, which is highly sensitive to oxygen and is usually part of a bifunctional ACS:CODH complex. Within these enzymes, multiple copies of different clusters exist in various oxidation and conformational states, often differing in the presence/absence of metal ions, for example, the Ni_P_ binding pocket is particularly prone to loss of the metal.^8,13^ These cumulative challenges result in spectroscopic data that are difficult to interpret. A recent study by Sarangi, Ragsdale, and co-workers used a highly active recombinant ACS-only enzyme to study methyl- and acyl-containing intermediate species for the first time, allowing for the trapping of a Ni^2+^–CH_3_ species which could be converted to a Ni^2+^–acyl species upon incubation with CO, confirming the viability of a step common to both the paramagnetic and diamagnetic pathways.^15^ The authors note that their results do not rule out the formation of a short-lived Ni^3+^–CH_3_ intermediate, as the presence of an in situ reductant (Ti^3+^ citrate) would rapidly reduce any Ni^3+^ to Ni^2+^ and prevent detection of any Ni^3+^ species, which highlights the challenges involved with interpretation of enzyme data.

The ambiguity surrounding nickel oxidation states has stimulated efforts toward preparing model complexes which replicate key features and reactivities of possible A-cluster intermediates involved in the biological mechanism.^9−11,23−26^ The Rauchfuss and Darensbourg groups demonstrated the ability to template multimetallic complexes starting from square-planar Ni^2+^ dithiolate complexes.^27−29^ Expanding on this work, Riordan, Matsumoto, Tatsumi, and co-workers demonstrated the production of thioesters from bimetallic complexes featuring Ni^2+^–CH_3_ and CO gas (a and b; Figure 1), presumed to proceed via Ni–acetyl intermediates.^9−11^ While the order of substrate binding to the A-cluster is debated,^16,20,21^ it is generally agreed that enzymatic migratory insertion to form Ni–acyl species occurs exclusively via the Ni^2+^ oxidation state, with one branch of the paramagnetic mechanism incorporating a ferredoxin-based electron shuttle to enable reduction of the proposed Ni^3+^–CH_3_ to Ni^2+^–CH_3_.^30^ Indeed, the few isolable monometallic Ni^3+^–CH_3_ complexes do not form the required Ni^3+^−acyl species upon exposure to CO, but instead release a methyl radical to afford the corresponding Ni^2+^–CO complex (c; Figure 1).^12,31^ In a recent approach, Shafaat and co-workers have used modified azurin proteins to spectroscopically probe several monometallic species relevant to the A-cluster featuring Ni–CO and Ni–CH_3_ moieties which are competent for generation of thioesters,^32^ and have identified an *S* = 1/2 Ni–CH_3_ species which the authors assign as Ni^3+^ with an “inverted” Ni–C bond (i.e., a cationic CH_3_ moiety).^33−35^ Such a species remains undetected in the enzyme itself but raises exciting questions about the nature of organometallic bonds accessible in natural systems.

Despite the significant achievements of previous A-cluster model complexes, there is a deficit of ligands capable of stabilizing bimetallic clusters in coordination environments and oxidation states relevant to the A-cluster. Bimetallic clusters featuring three-coordinate Ni^1+/0^ as well as organometallic Ni^1+^–CO and Ni^3+^–CH_3_ groups would mimic unexplored intermediates on the proposed catalytic cycle of the A-cluster. Further, such complexes would allow for exploration of the role of the Ni_D_ center, which is thought to remain Ni^2+^ and not form any organometallic intermediates during enzyme function; thus its role in the A-cluster remains an open question. Iron–sulfur clusters are responsible for electron transfer in many biological processes,^36^ and while the exact role of the [Fe_4_S_4_] moiety in the A-cluster is not well understood, it has been proposed to be exchange-coupled to the Ni_P_^1+^ site. This results in an overall *S* = 0 spin state for the A-cluster, which may prevent the study of some enzyme intermediates by electron paramagnetic resonance (EPR) spectroscopy.^37^ We hypothesized that by omitting the iron–sulfur cluster in the design of bimetallic complexes, we could isolate species such as those which occur transiently in the catalytic mechanism and study them in greater detail. To this end, we targeted the installation of an N-heterocyclic carbene (NHC) ligand in place of [Fe_4_S_4_]. NHCs are excellent ligands for stabilizing transition metals in both high and low oxidation states while offering tunable steric properties in order to kinetically stabilize reactive species.^38,39^ While the bioinorganic interest in NHCs was ignited by their compositional similarity to histidine, they have found widespread use as supporting ligands for many biological model complexes,^40−44^ including models of the nickel-containing enzyme CODH.^45^ In this work, we isolate the first bimetallic models of two hypothesized intermediates on the paramagnetic pathway of ACS function. Namely, an anionic {Ni^2+^Ni^1+^} cluster [**1**]^−^ featuring a three-coordinate nickel comparable to **A**_**red**_, and a {Ni^2+^Ni^1+^–CO} cluster [**2**]^−^ analogous to **A**_**NiFeC**_. We also investigated the competence of [**2**]^−^ for the generation of thioesters in a reaction analogous to the generation of the thioester acetyl coenzyme A by ACS.

## Results and Discussion

**Scheme 1:**
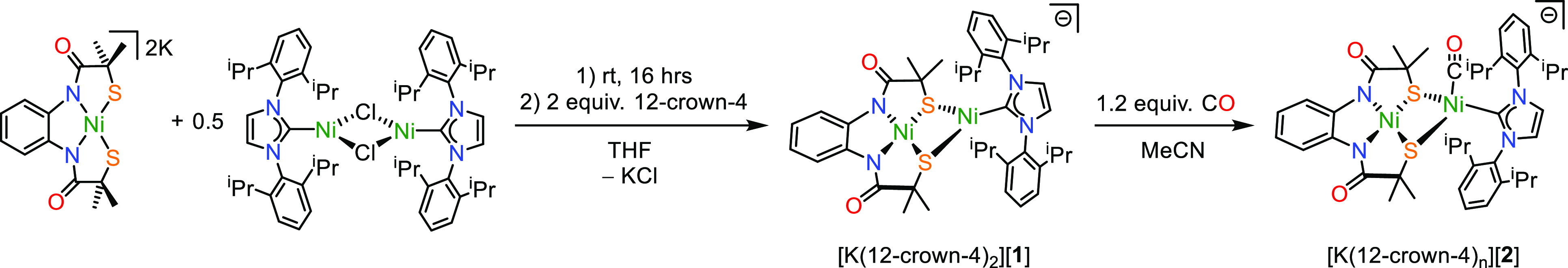
Synthesis of Cluster [K(12-crown-4)_2_][**1**] and the Binding of Carbon Monoxide to give [K(12-crown-4)_*n*_][**2**], *n* = 1 or 2 (See Discussion and Supporting Information for Details)

**Figure 2:**
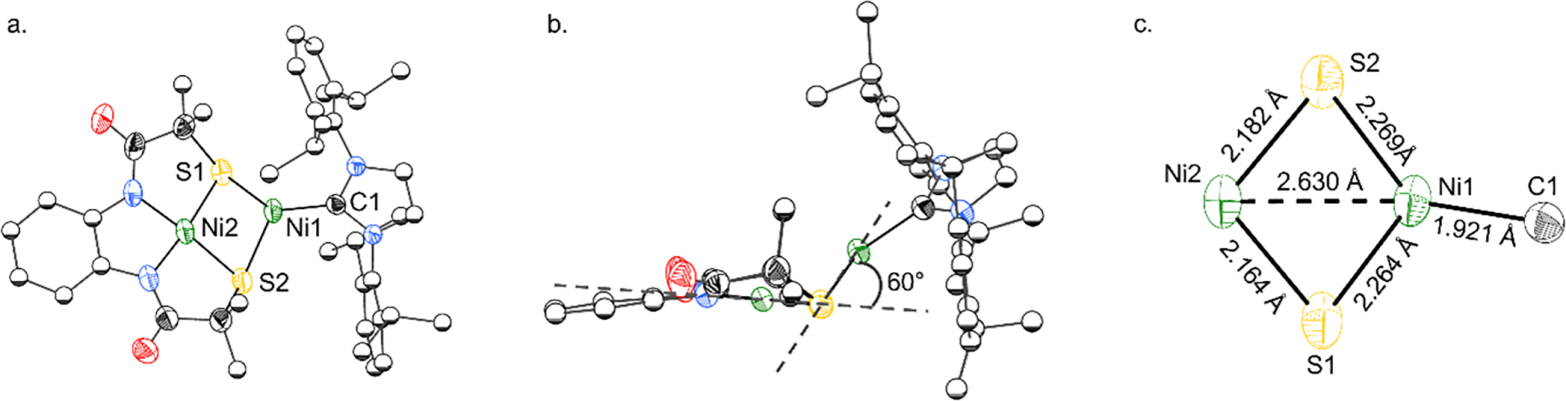
(a) Solid-state structure of the anion [**1**]^−^. (b) Side view of cluster [**1**]^−^ showing plane angle between binding pockets, defined by N,N,S,S and S,S,Ni1 planes. (c) Bond lengths for cluster core. In all cases, anisotropic displacement ellipsoids were depicted at 50% probability. [K(12-crown-4)_2_] cation and hydrogen atoms are omitted for clarity, and most ligand carbon atoms are displayed as spheres of arbitrary radius.

The condensation of the dianionic Ni^2+^ complex K_2_[LNi] (L = *N,N′*-1,2-phenylene-bis(2-sulfanyl-2-methylpropionamide))^46^ with half an equivalent of the Ni^1+^ complex {IPrNiCl}_2_ (IPr = 1,3-di(2′,6′-diisopropylphenyl)imidazolin-2-ylidene)^47^ (Scheme 1) generates the new bimetallic species [K(12-crown-4)_2_][**1**], which displays paramagnetically shifted and broadened NMR resonances (Figure S3). Single crystal X-ray diffraction revealed [**1**]^−^ to be an anionic {NiNi} cluster with a potassium ion sequestered by two equivalents of 12-crown-4 (Figure 2). The Ni1···Ni2 distance in **1** is 2.630(1) Å, too long to be a bond (sum of the covalent radii for Ni–Ni, Σ_Rcov_(NiNi) = 2.2 Å).^48^ The Ni2 is square planar, sitting within the N_2_S_2_ plane of the ligand, L (sum of angles 360°, where 360° indicates planarity), and Ni1 is coordinated by two thiolates of L in addition to one NHC. Square-planar nickel metalloligands featuring thiolates have been reported previously and exhibit hinge-like coordination modes, in which the second metal sits above the plane of the square-planar metalloligand.^49,50^ In [**1**]^−^, this hinge angle, defined by the N_2_S_2_ and S_2_Ni1 planes, is 60° (Figure 2b), resulting in Ni1 being displaced from the N_2_S_2_ plane by 1.37 Å. Surprisingly, Ni1 is three-coordinate despite crystallization from the coordinating solvent acetonitrile. The coordination environment around the Ni1 atom is distorted from planarity (the sum of bond angles about Ni1 is 353°). Although Y-shaped geometry (in which all angles about the metal center are 120°) is sterically favored in three-coordinate complexes,^51−54^ T-shaped geometry (where two angles are 90° and the third angle is 180°) can also be observed. In [**1**]^−^, the geometry about Ni1 is distorted toward T-shaped with an S–Ni–S angle of 91.4(2)° and S–Ni–C angles of 139.3(1)° and 122.5(1)°. Unrestrained T-shaped geometry (i.e., not enforced by a rigid ligand) is less commonly observed than Y-geometry; however it has been observed in a Ni^1+^ complex stabilized by a bulky β-diketiminate ligand, (nacnac)NiCO (nacnac = 2,4-bis(2,6-diisopropylphenylimido)pentyl), as well as in (dtbpe)NiCH_2_C(CH_3_)_3_ (dtbpe = 1,2-bis(diisopropylphosphino)ethane), and NiCl(IPr)_2_.^55−57^ In the former case, the geometric preference was rationalized through a degree of overlap between the metal and the carbonyl ligand, resulting in an overall stabilization of nickel d-orbitals.^55^ Computational analysis of [**1**]^−^ indicates that the pseudo-T-conformation is maintained in the gas phase (see Supporting Information Section 10) and is more stable than the lowest energy Y-conformer by 22 kcal mol^–1^ (Figure S18), which is a significant energetic difference for a minor geometric distortion. Analysis of the frontier molecular orbitals of [**1**]^−^ in the energetically favorable pseudo-T-conformation reveals a degree of orbital overlap in the SOMO-1 between the thiolate S1, Ni1, and C1, resulting in an orbital of π-symmetry that allows delocalization between the three atoms (Figure S23). Restraining the computational model to the Y-conformer eliminates this interaction and likely relates to the lower stability of this conformation. The structure of [**1**]^−^ is remarkably similar to that proposed for the **A**_**red**_ state of ACS, and it represents the best synthetic model to date by replicating the coordination environment around each Ni center. The **A**_**red**_ state of ACS has not been structurally characterized, however single crystal X-ray structures of the nickel-containing enzyme CODH (PDB: 1JJY)^58^ feature nickel in a T-shaped geometry. This unusual geometry has resulted in the proposal of a hydride invisible to protein crystallography;^59^ however, our results indicate that pseudo-T-shaped conformation can be energetically favorable for Ni^1+^ in a sulfur-rich coordination environment.

**Figure 3:**
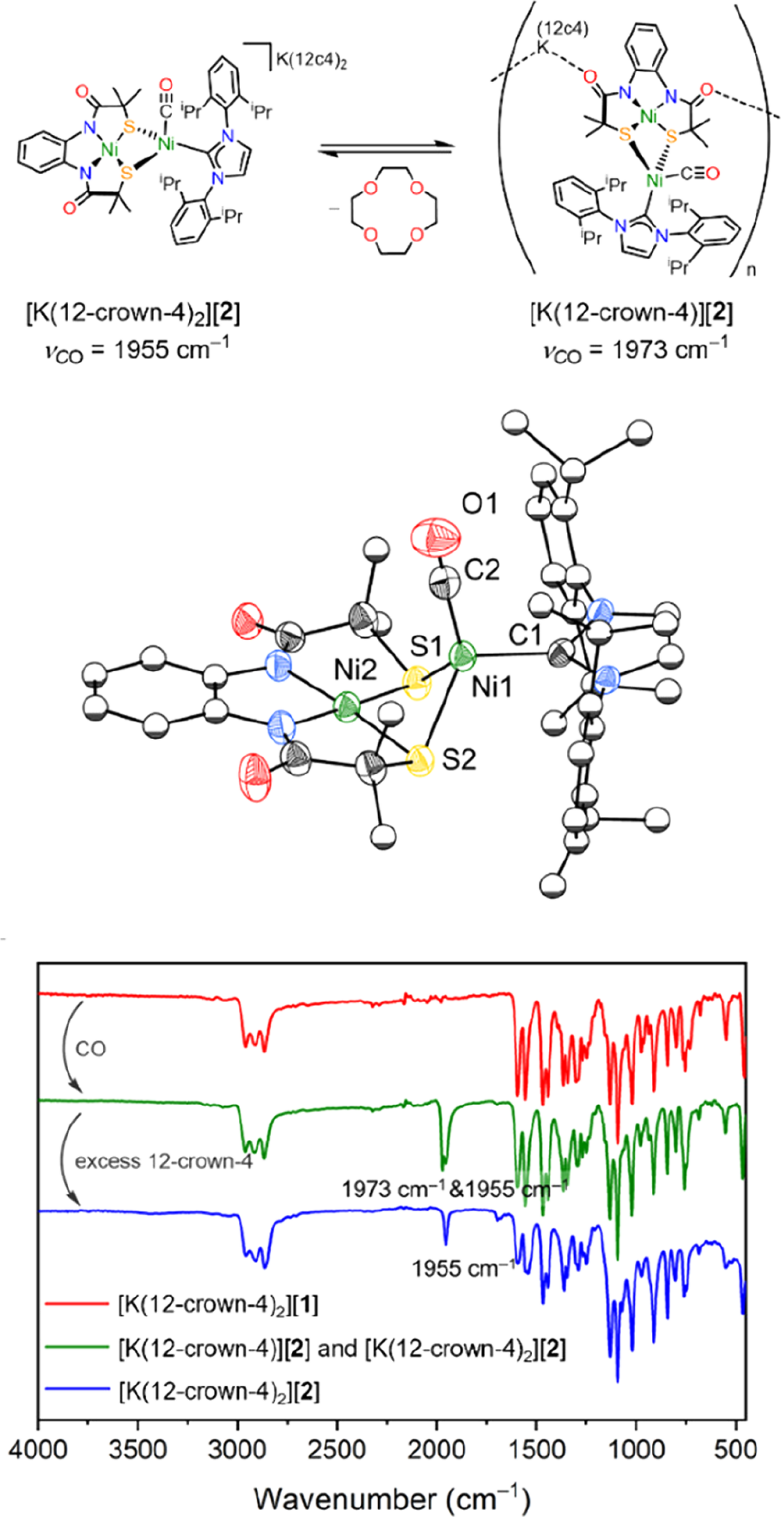
Top: Solution phase equilibrium for [**2**]^*–*^ shows the loss of 12-crown-4 from the potassium cation to form an ionic polymer. 12-crown-4 abbreviated as 12c4. Middle: Solid-state structure of [K(12-crown-4)][**2**]. Anisotropic displacement ellipsoids are depicted at 50% probability. K(12-crown-4) cation and hydrogen atoms are omitted for clarity, and most ligand carbon atoms are displayed as spheres of arbitrary radius. Bottom: FTIR spectra of [K(12-crown-4)_2_][**1**] (red), crystals containing [K(12-crown-4)_2_[**2**] and [K(12-crown-4)[**2**] (green), and [K(12-crown-4)_2_][**2**] only (blue).

Addition of a slight excess of carbon monoxide to a solution of [**1**]^−^ results in an immediate color change from yellow to purple, concomitant with the observation of a new paramagnetic species by ^1^H NMR spectroscopy (Figure S5). Crystals were obtained by layering an acetonitrile solution of the product with diethyl ether, and Fourier-transform infrared (FTIR) analysis of the crystalline material revealed two bands that are assigned to terminal CO stretches (1955 and 1973 cm^–1^, Figure 3; Bottom). Following dissolution of the crystalline material, addition of an excess of 12-crown-4, and evaporation to dryness, FTIR analysis of the dried (noncrystalline) solid reveals a species with a single band assigned as a CO stretch, with a frequency of at 1955 cm^–1^. Crystallization of this material resulted in recovery of the two stretching frequencies by FTIR, implying the presence of two polymorphs (Figure 3; Top), with terminal CO stretches of 1955 and 1973 cm^–1^, respectively.

Single crystal X-ray diffraction studies revealed two morphologies of crystals: red needles that were unsuitable for analysis, which we tentatively assign as o[K(12-crown-4)_2_][**2**] (Figure 3; Top), and red plates of sufficient quality for single crystal X-ray diffraction. Analysis of these plates revealed a 1D coordination polymer, [K(12-crown-4)][**2**], formed from the {NiNi} clusters bridged by [K(12-crown-4)] cations via carbonyl oxygen (Figure S16). A terminal carbonyl ligand is bound to the tetrahedral Ni1 site (Figure 3; Middle, τ_4_ = 0.793, where 1.0 indicates an ideal tetrahedral environment).^60^ Regarding the Ni–CO moiety, the structure reveals a Ni1–C2 bond length of 1.792(4) Å, with an almost linear Ni1–C2–O1 bond angle (178.5(3)°). Contrasting [**1**]^−^ and [**2**]^−^ reveals a modest elongation of the Ni1–S bond distances (2.264(1)/2.269(1) Å for [**1**]^−^ vs 2.333(1)/2.340(1) Å in [**2**]^−^). The Ni···Ni distance is reduced slightly upon coordination of CO in [**2**]^−^ to 2.582(1) Å (cf. 2.6303(4) Å in [**1**]^−^), though this still falls beyond a typical bonding interaction (∼2.2 Å).^48^ The plane angle increases in [**2**]^−^ (∠N_2_S_2_ and S_2_Ni1 plane = 108° vs 60° in [**1**]^–^), which increases the distance between Ni1 and the N_2_S_2_ plane (1.65 Å) (see Figure S17 for comparison). Ni1 in [**2**]^−^ also features a longer Ni1–C1 (1.977(3) Å) distance in comparison to the three-coordinate Ni1 in [**1**]^−^ (1.921(2) Å, respectively). The Ni2 remains square planar (sum of bond angles about Ni2 is 360°), while the Ni2–S bonds contract from 2.182(1)/2.164(1) Å in [**1**]^−^ to 2.154(1)/2.151(1) Å in [**2**]^−^. Further, the solid-state structure of [**2**]^−^ is similar to the CO-bound form of the A-cluster, which features similar Ni_P_–μ_2_S distances (2.31 and 2.28 Å), a comparable Ni–C–O angle (173°), a shorter Ni_P_–C_CO_ distance (1.63 Å), and is tetrahedral about Ni_P_ (τ_4_ = 0.791).^19^ Despite the observation of polymorphs ([K(12-crown-4)][**2**] and proposed [K(12-crown-4)_2_][**2**]) in the solid state, our data (EPR, NMR, FTIR, *vide infra*) indicate that in solution the anion [**2**]^−^ is consistent with the potassium cation sequestered by 12-crown-4 and/or by coordinating solvent. The different carbonyl absorptions observed in the FTIR spectra of crystalline samples of [**2**]^−^ are due to the formation of two polymorphs in the solid state only (which we believe is due to the presence or absence of one 12-crown-4 molecule), which differ in the location of the cation.

Despite the prevalence of carbonyl ligands in coordination chemistry, there are few structurally authenticated terminal Ni^1+^–CO complexes (Table S1).^61,62^ The tetrahedral, thioether-ligated (PhB(CH_2_S^t^Bu)_3_)NiCO features a significantly stronger CO bond with a stretching frequency of *v*_co_ = 1999 cm^–1^.^63,64^ The strong-field pincer complex (PNP)NiCO (PNP = 4,5-bis(diisopropylphosphino)-2,7,9,9-tetramethyl-9*H*-acridin-10-ide) has a CO stretching frequency of 1936 cm^–1^.^65^ [**2**]^−^ features two weak-field μ_2_-thiolate ligands and a strong-field NHC ligand, falling between the two previous examples and consistent with the general observation that increasing the electron density at the metal results in greater backdonation into the CO π*-orbital and thus weakening of the CO bond. The Shafaat group’s azurin-stabilized Ni^1+^–CO exhibits a stretching frequency of 1976 cm^–1^,^33^ while that of the ACS cofactor has been reported as 1998 cm^–1^,^16^ indicating that an all-sulfur environment results in less nickel-to-carbonyl backbonding compared to [**2**]^−^. Notably, the coordination of potassium cations to the ligand backbone of [K(12-crown-4)[**2**] (Figure 3) results in *v*_co_ = 1973 cm^–1^; blue-shifted by 18 cm^–1^ with respect to [K(12-crown-4)_2_[**2**] and moving it closer to the value observed for the cofactor. The importance of electrostatic interactions on enzyme catalysts, particularly around the active site, is well established.^66−68^ A computational study on iridium pincer complexes reported that changing the identity of a donor ligand (Si → Ge → Sn, C → B → Al → Ga → In) resulted in a change in carbonyl stretching frequency Δ*v*_co_ = ± 2–15 cm^–1^.^69^ [K(12-crown-4)_2_[**2**] and [K(12-crown-4)[**2**] demonstrate that interaction between a potassium cation and a distal part of the ligand, five bonds away from the spectroscopic probe (CO), can exert an effect on the CO stretching frequency comparable to changing the ligand directly bound to the metal center.

Density functional theory (DFT) calculations were performed to better understand the mechanism of binding of CO to [**1**]^−^. Structures of anions [**1**]^−^ and [**2**]^−^ were optimized at 298 K starting from the solid-state structure coordinates. The functional B3LYP and basis set def2-TZVP were used on all atoms with Grimme’s third dispersion correction factor (gd3).^70−72^ Unless otherwise specified, the calculations were performed with the application of a continuum solvation model to mimic the effect of the MeCN solvent. The calculations reproduced most of the experimental bond distances well (Table S4), although they overestimate the Ni···Ni distance observed in the polymeric [K(12-crown-4)][**2**] (calculated = 2.754; experimental = 2.6303(4) Å). This may in part be due to coordination of the potassium cation to [**2**]^−^. Despite this, the calculated CO stretching frequency after the application of a correction factor, *v*_COcalc_ = 1966 cm^–1^, is in excellent agreement with the experimental values (cf. 1953 for [K(12-crown-4)_2_][**2**], which features a fully sequestered cation and better resembles the calculated anion, and 1978 cm^–1^ for the cation-coordinated [K(12-crown-4)][**2**]) and gives us confidence in our method to reproduce experimental parameters. Performing a relaxed surface scan (BP86/def2-SVP) upon elongation of the Ni1···CO bond indicates that association of CO to nickel is barrierless (Figure S19).^73,74^ Multiple possible transition states were found by contractively scanning the Ni1···CO distance; however, the intensity of the imaginary frequencies were <91 cm^–1^ in all cases; too small to be true transition states. Indeed, the barrier to CO coordination is small (up to 4.4 kcal mol^–1^; Figure S20) and associated with rotation about the Ni1–C_NHC_ bond in [**1**]^−^ to allow the access of CO to the Ni1 binding site. In all the scans performed, the CO approaches nickel in a nonlinear fashion (Ni1–C2–O1 = 114°), consistent with interaction between the occupied 3d_*z*_^2^-orbital of Ni1 and the canonical π*-orbital of CO (Figure S21). The overall reaction ([**1**]^−^ + CO → [**2**]^−^) was found to be exergonic by 6 kcal mol^–1^, indicating that CO binds only weakly to Ni^1+^.

The conversion of [**1**]^−^ to [**2**]^−^ upon the addition of CO is accompanied by a color change from yellow to purple. The optical absorption spectrum of [**1**]^−^ displays a strong absorbance at 405 nm, a shoulder at 448 nm, and a weaker absorbance at 654 nm (Figure S11). Upon conversion to [**2**]^−^, these features significantly reduce in intensity, and a new absorbance at λ_max_ = 562 nm appears, accounting for the purple color of the solution. Time-dependent DFT (TD-DFT) calculations performed on [**2**]^−^ produce a calculated UV/vis spectrum in excellent agreement with the experimental data (Figure S24). The calculated excitation at 564 nm (State 9) is dominated by the excitation of a β electron from 222β, the SOMO-1, to 223β, the LUMO. This transition can be described as a charge transfer from the ligand nitrogen atoms and the 3d_*xz*_ of the square-planar Ni2 (222β) into the Ni1 3d_*xy*_ orbital, which is bonding with respect to the Ni1–C2 bond, and the π*-antibonding orbital of the CO ligand. Indeed, all the transitions contributing to the absorbances at 573, 564, and 555 nm involve charge transfer from Ni2 d-orbitals to the CO π*-antibonding orbital, and most also incorporate charge transfer from the ligand, L (Figures S25 and S26). Conceptually similar mixed metal-to-ligand-to-ligand charge transfers (MMLLCT) have been observed for square-planar group 10 (Ni, Pd, and Pt) complexes, including those with thiolate ligands.^75,76^ In [**2**]^−^, Ni2 facilitates the transfer of an electron from L into the Ni1–C2 bonding and antibonding orbitals as well as the antibonding orbital of the carbon monoxide ligand, which will result in an elongation of the C–O bond in the excited state.

**Figure 4:**
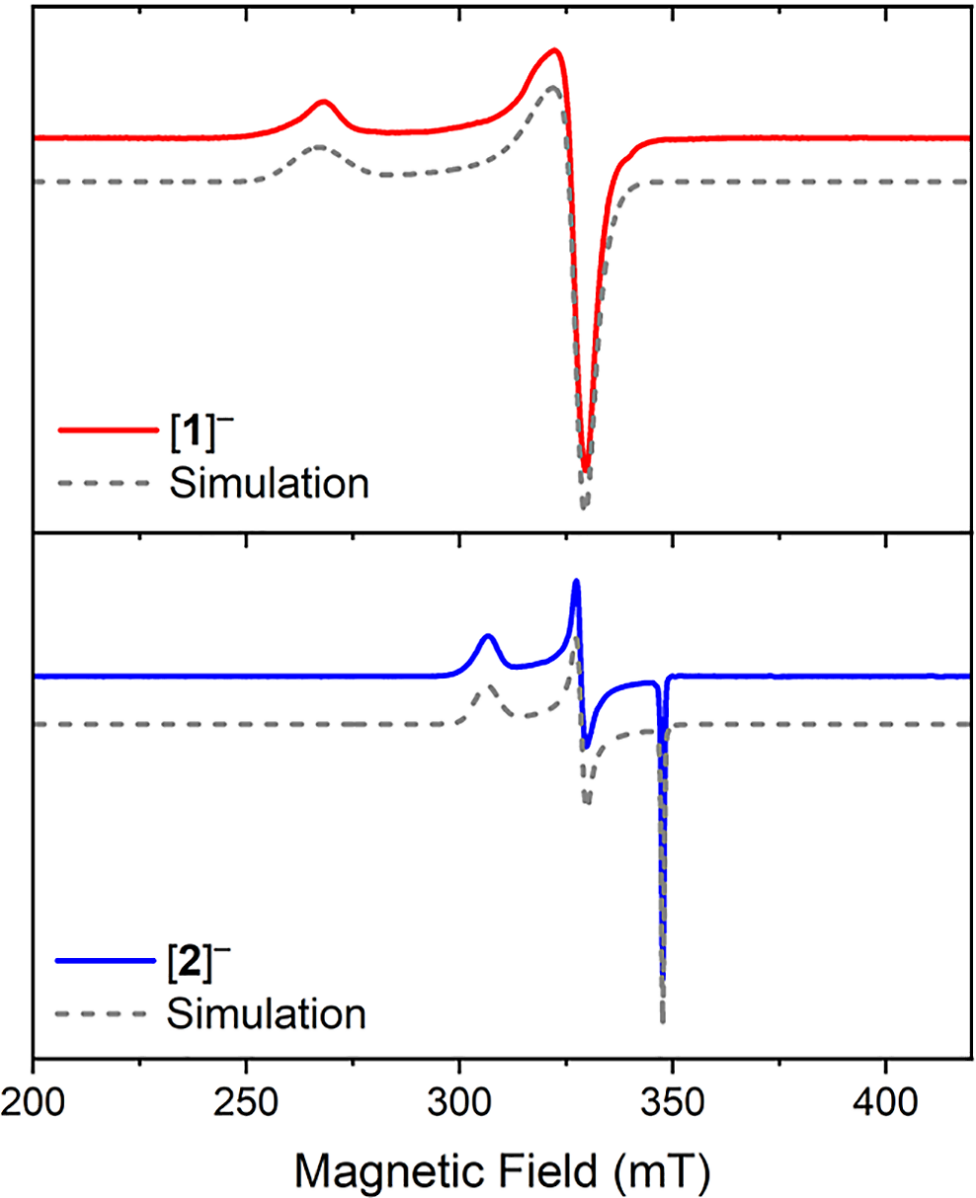
Frozen-matrix X-band CW-EPR spectra of 2-methyltetrahydrofuran solutions of [**1**]^*–*^ (red trace, top panel) and [**2**]^*–*^ (blue trace, bottom panel) recorded at 93 and 25 K, respectively (solid lines) together with the simulations (dashed lines). The measurement conditions and the fitting parameters are reported in the Supporting Information.

EPR analysis of a frozen 2-methyltetrahydrofuran solution of [**1**]^−^ displays a pseudoaxial signal, which can be modeled as an *S* = 1/2 center, with *g*_1_ = 2.538, *g*_2_ = 2.071, and *g*_3_ = 2.062 (Figure 4, see also Supporting Information Section 8.0). This is consistent with the square-planar Ni2^2+^ site being *S* = 0 and, therefore, EPR silent. Although DFT calculations successfully reproduced the trend in *g*-values of *g*_1_ > *g*_2_, *g*_3_ in both the T- and Y-shaped conformers, the magnitude of *g*_1_ was consistently underestimated (Table S3) due to difficulty in reproducing the covalency of Ni–S bonds (see Supporting Information Section 10.3).^77^ The frozen-matrix X-band EPR spectrum of [**2**]^−^ is rhombic and can be simulated as a single *S* = 1/2 signal with *g*_1_ = 2.267, *g*_2_ = 2.114, and *g*_3_ = 1.997. An *S* = 1/2 spin state is assigned based on the observation of microwave power saturation effects (*P*_1/2_ = 0.7 mW at 25 K, see Supporting Information). DFT calculations (B3LYP-Def2-TZP-gd3) align with the magnetic properties of [**2**]^−^, with calculated *g*_1_ = 2.242, *g*_2_ = 2.148, and *g*_3_ = 2.018 indicating a pseudotetrahedral Ni^1+^ environment.

DFT calculations on [**1**]^−^ and [**2**]^−^ reveal highly localized spin density at the three-coordinate and CO-coordinated Ni sites, respectively (89% for [**1**]^−^; 85% for [**2**]^−^), with only a small amount found on the square-planar nickel (2 and 6%, respectively; Figure S22). In [**1**]^−^, there is an asymmetric distribution of density between the two bridging thiolate ligands, with <1% on S1 and 9% on S2, which is involved in π-symmetry interactions with Ni1. [**2**]^−^ displays a small amount of spin density on the ligands directly coordinated to the tetrahedral nickel, with 3% on each S and 6% delocalized onto the carbon atom of the CO ligand. There is less than 1% spin density on the NHC carbonic carbon and nitrogen atoms in both cases, which is consistent with the absence of ^14^N hyperfine coupling in the EPR spectrum and indicates that the NHC ligand does not play a significant role in delocalizing spin density in these complexes. This is unusual given the propensity for NHCs to stabilize unpaired electrons in a wide variety of transition metal complexes, and indicates that the NHC is primarily for structural support.^78^

**Figure 5:**
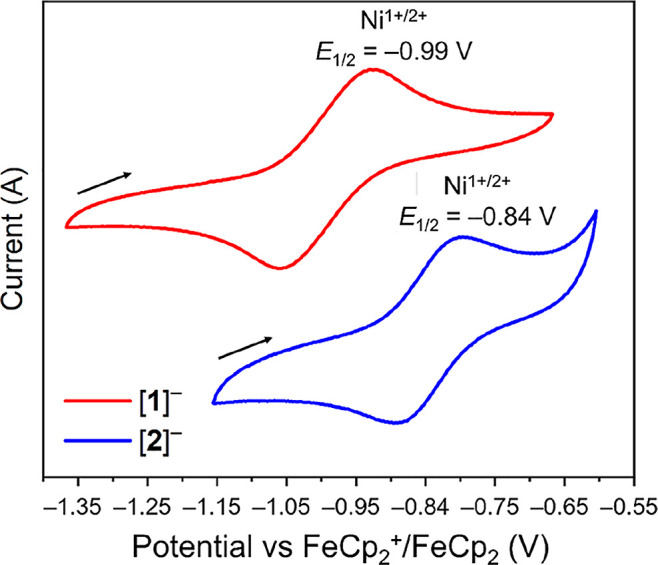
Cyclic voltammograms of [**1**]^*–*^ and [**2**]^*–*^ showing the Ni^1+/2+^ redox couple. Solvent: MeCN, 0.1 M [^*n*^Bu_4_N]PF_6_, scan rate 100 mV s^–1^, *y*-axis scaled for clarity.

The cyclic voltammogram of [**1**]^−^ (Figure 5) shows one quasi-reversible redox event at −0.99 V (vs FeCp_2_^+^/FeCp_2_) (3.9 mM, MeCN), tentatively assigned as the Ni^1+^/^2+^ redox couple for the 3-coordinate nickel center, with full chemical reversibility (Figure S13). This assignment is consistent with reported potentials for the Ni^1+/2+^ redox couple in well-defined complexes. There are relatively few accessible, fully (chemically) reversible Ni^1+/2+^ redox couples featuring three-coordinate Ni, but those documented have potentials of −1.25 V for (1,2-bis(di*tert*-butylphosphino)ethane)Ni(CH_2_CMe_3_),^79^ and −0.90 V for (1,2-bis(di*tert*-butylphosphino)ethane)Ni(NH(2,6-(CHMe_2_)_2_C_6_H_3_).^80^ Examples of electron-rich Ni complexes stabilized by π-withdrawing NHC ligands include the two-coordinate IPrNi[NH(2,6-di-isopropylphenyl)], where the Ni^1+/2+^ couple is observed at 0.84 V (vs FeCp_2_/FeCp_2_^+^),^81^ and the formally Ni^0^ Ni(TIMEN^t^Bu) (TIMEN^*t*^Bu = tris[2-(3-*tert*-butylimidazol-2-ylidene)ethyl]amine), where oxidation to Ni^1+^ occurs at −2.50 V, followed by a second oxidation from Ni^1+^ to Ni^2+^ at −1.09 V (vs FeCp_2_/FeCp_2_^+^).^82^ Several further examples are given in Table S2 and while these cannot be considered like-for-like comparisons given differences in ligand set, geometry, and conditions, they nonetheless demonstrate that the measured Ni^1+/2+^ value for [**1**]^−^ is appropriate for a low-coordinate Ni^1+^ center.

Upon coordination of CO to the nickel center ([**2**]^−^, 2.4 mM in MeCN), this Ni^1+^/Ni^2+^ redox couple undergoes an anodic shift to −0.84 V. This positive shift is attributed in part to Ni–CO π backbonding, which acts to reduce the overall electron density at the Ni center and makes the metal center more difficult to oxidize. The extended cyclic voltammogram of [**2**]^−^ (Figure S12) displays irreversible features at −1.55 and −1.86 V, which are potentially associated with the formation of Ni^0^ species and are not present in the cyclic voltammogram of [**1**]^−^.

Chemical oxidation of [**2**]^−^ with ferrocenium hexafluorophosphate in MeCN solution results in the rapid formation of a diamagnetic species by ^1^H NMR spectroscopy with concomitant loss of the absorbance corresponding to Ni–CO in the FTIR spectrum. Analogous chemical oxidation of [**1**]^−^ results in a species with an identical NMR signature, and this species does not show any reactivity toward CO. Attempts to isolate the oxidized product in either case were unsuccessful. However, these results suggest that upon oxidation of [**2**]^−^, an irreversible loss of CO from Ni^2+^ occurs. Further, while previous reports have demonstrated the feasibility of a β-diketiminate Ni^0^–CO complex in thioester synthesis,^83^ our results for complexes [**1**]^−^ and [**2**]^−^ indicate that their biologically inspired ligand scaffold is not capable of stabilizing the Ni^0^ oxidation state, despite the presence of a π-accepting NHC ligand suitable for the stabilization of a low oxidation state nickel. The A-cluster features no obvious π-accepting ligands, and it seems unlikely therefore that an all-sulfur environment is able to stabilize such a reduced metal site.^84^ However, it should be noted that steric and electrostatic interactions in the enzyme may enable a lower oxidation state to be reached.^85^ Nevertheless, our results suggest that biology utilizes Ni^1+^ due to its ability to bind CO while Ni^2+^ cannot, and this Ni^1+^ can be stabilized by the binding pocket, which is not the case for Ni^0^. Further, the weak binding of CO (Δ*G* = −6 kcal mol^–1^) and barrierless coordination result in a small energetic span, making it an ideal step for catalysis.^86^

**Scheme 2:**
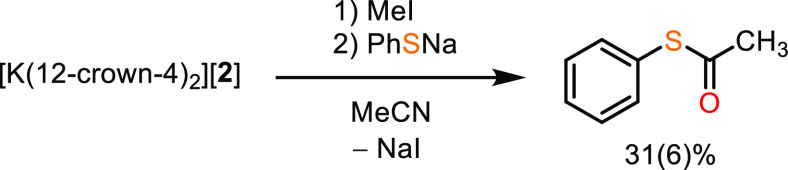
Formation of S-Phenyl Thioacetate from [K(12-crown-4)_2_][**2**]

Finally, we sought to assess whether complex [**2**]^−^ could perform reactions similar to the A-cluster, specifically with respect to the formation of thioesters (Scheme 2). Treatment of [**2**]^−^ with methyl iodide followed by sodium thiophenolate in MeCN afforded the corresponding thioester in 31(6)% yield, quantified by gas-chromatography/mass-spectrometry (GC/MS) (Supporting Information, Section 3.3). Lee and co-workers studied the transformation of (PNP)Ni^1+^–CO into (PNP)Ni^2+^–acetyl by addition of methyl iodide, noting that the first step is a reduction of ICH_3_ by (PNP)Ni^1+^–CO, yielding a (PNP)Ni^2+^–I and a methyl radical. The methyl radical then reacts with remaining (PNP)Ni^1+^–CO to form (PNP)Ni^2+^–acetyl.^23,24,87^ We hypothesize that a similar mechanism is active for [**2**]^−^ and limits the maximum yield of S-phenyl thioacetate to 50%. The ability to produce thioesters demonstrates that [**2**]^−^ is not only a structural mimic of the **A**_**NiFeC**_ state of the A-cluster, but capable of functioning analogously to ACS. This observation experimentally demonstrates that complexes like **A**_**NiFeC**_ are competent for thioester production, directly mimicking the chemistry of the ACS enzyme, and support the hypothesis that the **A**_**NiFeC**_ state is a feasible catalytic intermediate.^16^

## Conclusions

This study outlines the preparation of two heteroleptic, mixed valence {Ni^2+^Ni^1+^} clusters relevant to the enzyme ACS and their detailed characterization using spectroscopic and computational analyses. The structure of cluster [**1**]^−^ demonstrates that three-coordinate Ni^1+^ can be thermodynamically more stable in a pseudo-T-shaped geometry over the sterically preferred Y-shape. As there are no crystal structures of the **A**_**red**_ state of ACS, [**1**]^−^ provides a unique opportunity to study the reactivity of such species and provides spectroscopic signatures useful for those studying the enzymes. Paramagnetic [**1**]^−^ binds CO at Ni^1+^, yielding paramagnetic [**2**]^−^, which structurally is very similar to the reported crystal structure of CO-bound ACS. The reaction of [**1**]^−^ + CO → [**2**]^−^ mimics the enzymatic conversion of **A**_**red**_ to **A**_**NiFeC**_, and it is the first demonstration of its viability in a bimetallic model complex. Calculations indicate that CO is weakly bound to the Ni^1+^ in [**2**]^−^, which, in combination with the barrierless coordination of CO, results in a small energetic span, making this an ideal step for hypothetical catalysis. The CO of [**2**]^−^ is irreversibly lost upon oxidation to Ni^2+^ and the ligand scaffold is not able to support Ni^0^ despite the presence of π-accepting NHC and CO ligands, implying that Ni^1+^ is ideal for binding CO in a biologically relevant coordination environment. Finally, [**2**]^−^ can convert the bound CO into a thioester upon addition of a methyl cation and thiolate, analogous to the function of ACS, which supports the hypothesis that the **A**_**NiFeC**_ state is an intermediate during ACS function.

## Supporting Information Available

Procedures and characterization data, NMR, EPR, Mass, UV/vis, and FTIR spectra, cyclic voltammetry data, single crystal X-ray diffraction figures, and computational details (PDF)
