# Supplementary material for: Mixed Valence {Ni2+Ni1+} Clusters as Models of Acetyl Coenzyme A Synthase Intermediates
Source: J Am Chem Soc. 2024 Jul 18;146(30):21034–43. doi: 10.1021/jacs.4c06241 (PMC11295191; doi:10.1021/jacs.4c06241)
Supplement: Supplementary file 1 — ja4c06241_si_001.pdf [file ja4c06241_si_001.pdf]

Supplementary Information

Mixed valence {$Ni^{2+}Ni^{1+}$} clusters as models of Acetyl Coenzyme A Synthase Intermediates

Daniel W. N. Wilson,*[a,b] Benedict C. Thompson,[a] Alberto Collauto,[c] Reagan X. Hooper,[d] Caroline E. Knapp,[b] Maxie M. Roessler,*[c] Rebecca A. Musgrave*[a]

[a]Department of Chemistry, King's College London, 7 Trinity Street, London, SE1 1DB, UK.

[b]Department of Chemistry, University College London, 20 Gordon Street, London, WC1H 0AJ, UK.

[c]Department of Chemistry and Centre for Pulse EPR Spectroscopy, Imperial College London, 82 Wood Lane, London, W12 0BZ, UK.

[d]Stanford PULSE Institute, SLAC National Accelerator Laboratory, Menlo Park, California 94025, USA

*email: dan.wilson@ucl.ac.uk; m.roessler@imperial.ac.uk; rebecca.musgrave@kcl.ac.uk

1.0. Supplementary Figure 2
2.0. General considerations 3
3.0. Synthetic procedures 5
3.1. Synthesis of [K(12-crown-4)$_2$][IPrNiNi(L)] ([K(12-crown-4)$_2$][**1**]). 5
3.2. Synthesis of [K(12-crown-4)$_1$][IPrNi(CO)Ni(L)] ([K(12-crown-4)$_1$][**2**]) and [K(12-crown-4)$_2$][IPrNi(CO)Ni(L)] ([K(12-crown-4)$_2$][**2**]). 6
3.3. Generation of S-Phenyl thioacetate 7
4.0. ^{1}H NMR spectra 9
5.0. FTIR spectra of metal complexes 11
6.0. UV-visible absorption spectra 13
7.0. Electrochemistry 14
8.0. EPR Spectroscopy 17
9.0. Crystallographic data 19
10.0. Computational Details 23
10.1. Thermodynamics from DFT calculations 26
10.2. Time-dependent density functional theory (TDDFT) 30
10.3. EPR Calculations 36
10.4. DFT Coordinates 38
11.0. References 50

1.0. Supplementary Figure

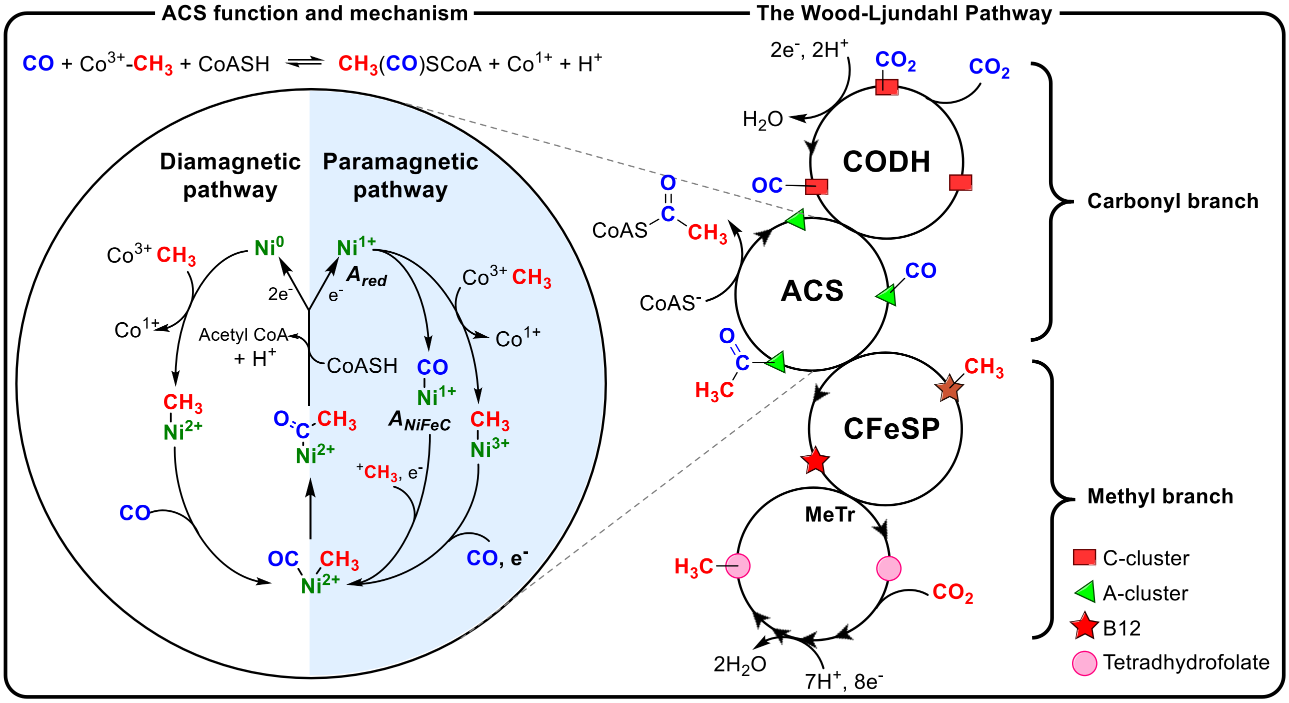

Figure S1. Schematic overview of the enzymes on the Wood-Ljundahl pathway (right) adapted from an image on the Drennan group website[1] and a summary of the proposed diamagnetic and paramagnetic reaction pathways (left) outlining key organometallic intermediates. All shown reactivity is at the Ni_P site and the rest of the cofactor omitted for clarity. ACS = acetyl coenzyme A synthase, CODH = carbon monoxide dehydrogenase, CFeSP = corrinoid iron-sulfur protein, MeTr = methyltransferase.

2.0. General considerations

All reactions and product manipulations of molecular species were carried out under an inert atmosphere of dinitrogen or argon using standard Schlenk line or glovebox techniques (MBraun glovebox MB150G-B maintained at <0.1 ppm H_2O and <0.1 ppm O_2), unless otherwise stated. Glassware for metalation reactions was oven dried at 200 °C overnight prior to use. Dry THF, diethyl ether and hexanes were obtained from a Grubbs-type solvent system employing alumina columns, stored over 4 Å molecular sieves, and sparged with N_2 prior to use. Acetonitrile was sparged with argon and stored over activated 3 Å molecular sieves for 4 days (sieves replaced after 48 hours) prior to use. MeCN-d_3 was purchased from Sigma-Aldrich, distilled from calcium hydride, and stored over 3 Å molecular sieves. Celite 521 was obtained from Fluorochem and heated to 200 °C for 16 hr prior to use. All solvents were stored in glove boxes over molecular sieves. Benzene-d_6 and THF-d_8 were dried over multiple rounds of 4 Å molecular sieves and degassed by freeze-pump-thawing prior to use. K_2[LNi] (L = *N*,*N'*-1,2-phenylene-bis(2-sulfanyl-2-methylpropionamide)) and {IPrNiCl}$_2$ (IPr = 1,3-di(2′,6′-diisopropylphenyl)imidazolin-2-ylidene) were synthesized according to literature procedures.[2,3] Ferrocenium hexafluorophosphate, sodium thiophenolate, and *S*-phenyl thioacetate were purchased from Sigma Aldrich and used as received. Methyl iodide was purchased from fluorochem and vacuum distilled and stored over activated alumina before use.

^{1}H NMR spectra were recorded at ambient temperature on a Bruker NMR 400 MHz spectrometer. All spectra are referenced to the most downfield residual solvent resonance. In all ^{1}H NMR spectra of paramagnetic species, backward linear prediction from 0 to 15 data points was employed to remove baseline distortion, phase correction was addressed manually, and an ablative baseline correction applied ^{1}H NMR spectra were collected between +250 and –250 ppm to observe signals at both low and high field (number of scans 2048).

X-ray crystallography: Suitable single crystals of [K(12-crown-4)$_2$][**1**] and [K(12-crown-4)$_1$][**2**] were coated with Fomblin® Y oil, and mounted onto a Hampton Research nylon loop (20 µm thickness, 0.3 mm diameter). All experiments were performed at 150 K using Cu Kα radiation (λ = 1.54184 Å). Measurements were made using a twin-source SuperNova diffractometer with a micro-focus Cu Kα X-ray beam (50 kV, 0.8 mA), an Atlas (135 mm CCD) detector. Cell refinement, data collection and data reduction for all experiments were performed using Rigaku CrysAlisPro 1.171.40.57a. Crystal structures were solved and refined by least-squares within the Olex2 program suite[4] using the ShelXT structure-solution program[5] and the ShelXL 2014 refinement program. Positions of hydrogen atoms were identified as the strongest peaks in Fourier difference maps and were either refined when appropriate or constrained with AFIX commands within SHELX. Crystallographic details are provided in Section 9.

UV/Visible absorption spectra were recorded on an Agilent Technologies Cary Series UV-Vis spectrophotometer. Samples were dissolved in acetonitrile and placed in a sealed quartz cuvette suitable for air- and moisture-sensitive compounds. ATR-IR spectra were recorded on a Shimadzu IRAffinity-1S Fourier Transform Infrared Spectrophotometer.

3.0. Synthetic procedures

3.1. Synthesis of [K(12-crown-4)$_2$][IPrNiNi(L)] ([K(12-crown-4)$_2$][**1**]).

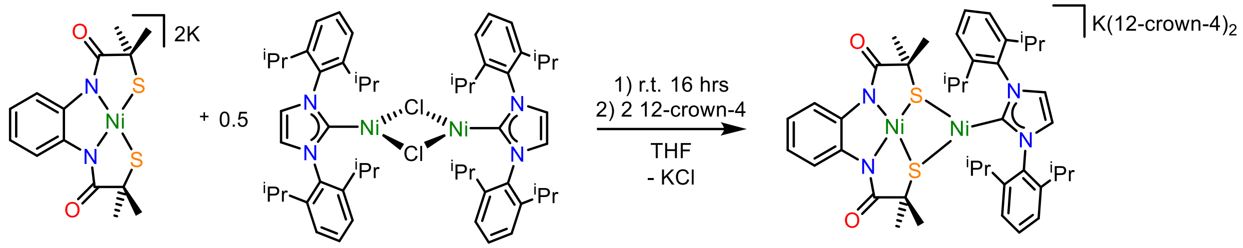

Inside a glovebox, $[IPrNiCl]_2$ (200 mg, 0.208 mmol) was dissolved in THF (5 mL). Separately, K_2[LNi] (202 mg, 0.416 mmol) was added to a 25 mL vial containing a stir bar and THF (5 mL) was added. The reaction was stirred for 10 minutes. To this stirring suspension, the solution of $\{IPrNiCl\}_2$ was added dropwise. The red suspension was stirred overnight at room temperature and filtered through celite to remove KCl salts. The THF was removed under reduced pressure, and the resulting red solid taken up into the minimal amount of MeCN (2 mL). 12-Crown-4 (147 mg, 0.832 mmol) was added with stirring and the mixture was stirred for 30 minutes. Highly air- and moisture-sensitive bright red crystals of [K(12-crown-4)$_2$][**1**] suitable for single crystal X-ray diffraction were grown by slow diffusion of Et_2O into a MeCN solution of the product at room temperature (310 mg, 60%).

^{1}H NMR (400 MHz, MeCN-*d*$_3$) δ 17.7 (1H), 10 (1H), 7.9 (4H), 7.4 (4H, IPr aromatic CH), 6.8 (1H, L aromatic CH), 6.5 (2H, L aromatic CH), 5.8 (2, IPr backbone CH), 4.62 (23H, 12-crown-4), 2.93 (36H, 12-crown-4, L CH_3, IPr CH_3 groups), –2.4 (br, 7H).

IR (ATR, neat, cm^{-1}): 2956, 2904, 2866, 1596, 1555, 1469, 1441, 1367, 1340, 1319, 1246, 1132, 1089, 1021, 974, 908, 843, 800, 753, 548, 458.

UV-vis (acetonitrile, nm): 405, 448, 654.

Elemental analysis calcd for $C_{57}H_{84}KN_4Ni_2O_{10}S_2$: C, 56.77; H, 7.02; N, 4.65. Found: C, 57.12; H, 7.21; N, 4.51.

3.2. Synthesis of [K(12-crown-4)$_1$][IPrNi(CO)Ni(L)] ([K(12-crown-4)$_1$][2]) and [K(12-crown-4)$_2$][IPrNi(CO)Ni(L)] ([K(12-crown-4)$_2$][2]).

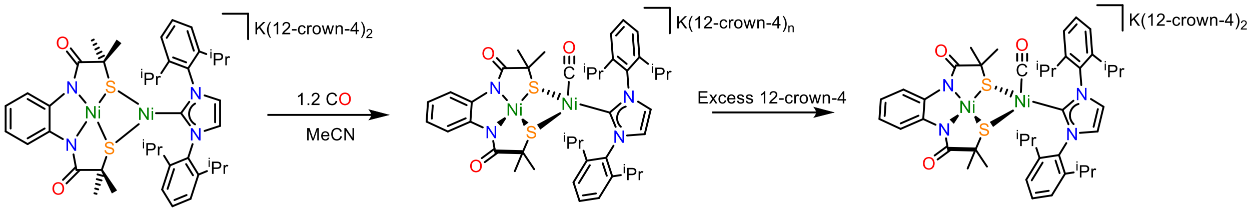

[K(12-crown-4)$_2$][**1**] (20 mg, 0.016 mmol) was dissolved in MeCN (0.5 mL) in a long, thin Schlenk flask. The solution was degassed by three freeze-pump-thaw cycles. Under a strictly inert atmosphere at room temperature, a slight excess of CO (1.2 equivalents, 2.1 mL at r.t.:1 Bar) was slowly bubbled through the solution using a needle and syringe. The solution color immediately changed from light orange to purple. Dark red crystals of [K(12-crown-4)$_1$][**2**] suitable for single crystal X-ray diffraction were grown by slow diffusion of Et_2O into a MeCN solution of the product at room temperature (16 mg, >78%, with error associated with yield due to loss of some 12-crown-4 in the product). Dissolution of the crystals in MeCN and addition of excess 12-crown-4, followed by removal of volatile materials, resulted in a dark red powder. Elemental analysis of this powder is consistent with the composition [K(12-crown-4)$_2$][**2**].

Performing the experiment with excess CO results in a complex mixture of products forming, accompanied by a colour change to light blue. NMR, IR, and UV-vis spectra of this solution indicated [K(12-crown-4)$_n$][**2**] to be the major product, in addition to further unidentified CO containing species.

^{1}H NMR (400 MHz, MeCN-d_3) δ (ppm) 40.5 (2H), 23 (2H), 9.54 (ligand, L, decomp product <5%), 8.4 (2H), 7.8 (2–3H), 7.2 (ligand L, decomp product <5%), 6.55 (2H), 3.8–5.5 (m, 21H broad singlet and 12-crown-4 protons), 2.74 (12-crown-4), 2.08 (2H), 1.49 (8H), 0.3 (4H), –2.5 (4H).

IR (ATR, neat, cm^{-1}): 2962, 2913, 2862, 1953 (ν_{CO}), 1123, 1595,1541,1469,1441, 1363, 1344, 1289, 1246, 1132, 1093, 1021, 974, 910, 843, 802, 761, 747, 548, 464.

UV-vis (acetonitrile, nm): 405 (weak), 448 (weak), 562, 654 (weak).

Elemental analysis calcd for $C_{58}H_{84}KN_4Ni_2O_{11}S_2$: C, 56.46; H, 6.86; N, 4.54. Found: C, 55.12; H, 6.11; N, 4.34.

3.3. Generation of S-Phenyl thioacetate

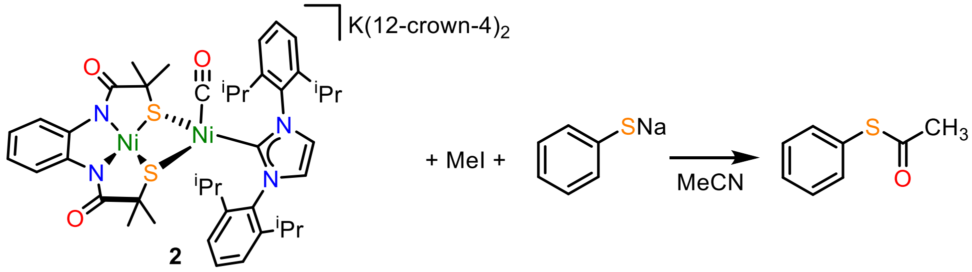

General procedure: Inside a glovebox, [K(12-crown-4)$_1$][**2**] (10 mg, 0.008 mmol), was dissolved in MeCN (0.5 mL). To this, MeCN (0.1 mL) was added followed by dropwise addition of a 20 mM solution of methyl iodide in MeCN (0.4 mL). The solution was stirred for 30 seconds. After this time, a 40 mM solution of sodium thiophenolate with solubilizing 12-crown-4 (0.1 mL) was added and the reaction stirred overnight. The reaction was removed from the glovebox and distilled to remove any salts and residual metal complexes. The resulting colourless distillate solution was diluted by a factor of 10 and analysed by GCMS, which was calibrated to external standards of S-phenyl thioacetate. Concentration of S-phenyl thioacetate: 0.30(2) mg mL^{-1}; 31% yield).

With reductant CoCp$_2$: the above procedure was modified to replace 0.1 mL of MeCN with 0.1 mL of an 80 mM solution of $CoCp_2$ in MeCN. Concentration of S-phenyl thioacetate: 0.33(2) mg mL^{-1}; 32% yield).

Control: control was prepared via the above procedure with 0.5 mL of MeCN replacing the solution of [K(12-crown-4)$_1$][**2**]. Concentration of S-phenyl thioacetate: Not detectable.

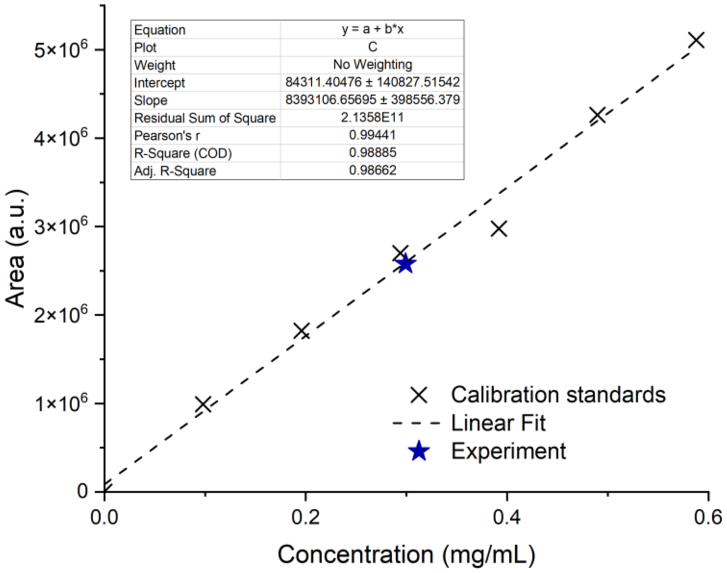

Figure S2. GC/MS calibration curve for the quantification of S-phenyl thioacetate.

4.0. ^{1}H NMR spectra

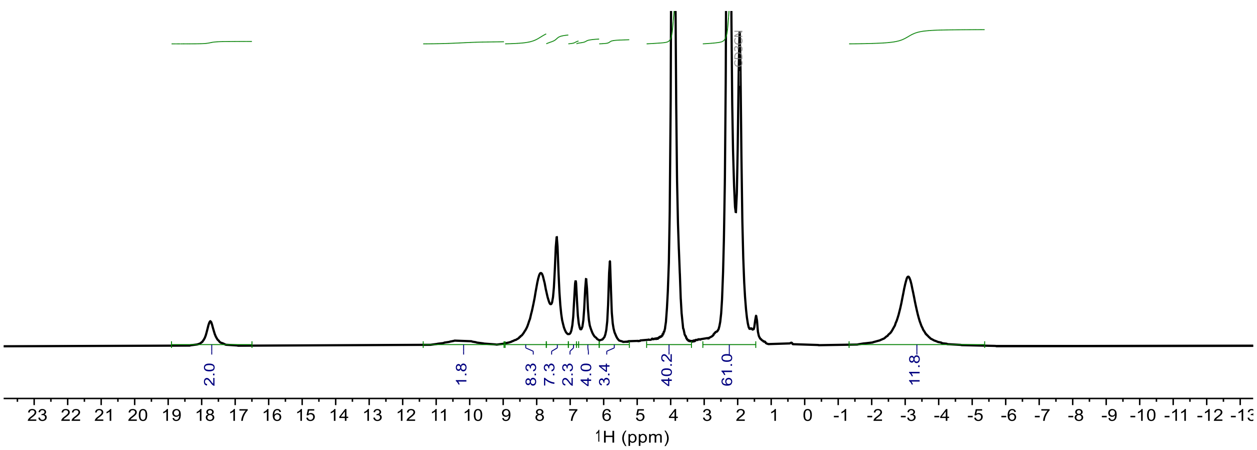

Figure S3. ^{1}H NMR (400 MHz) spectrum of [K(12-crown-4)$_2$][**1**] in MeCN-d_3 at 298 K.

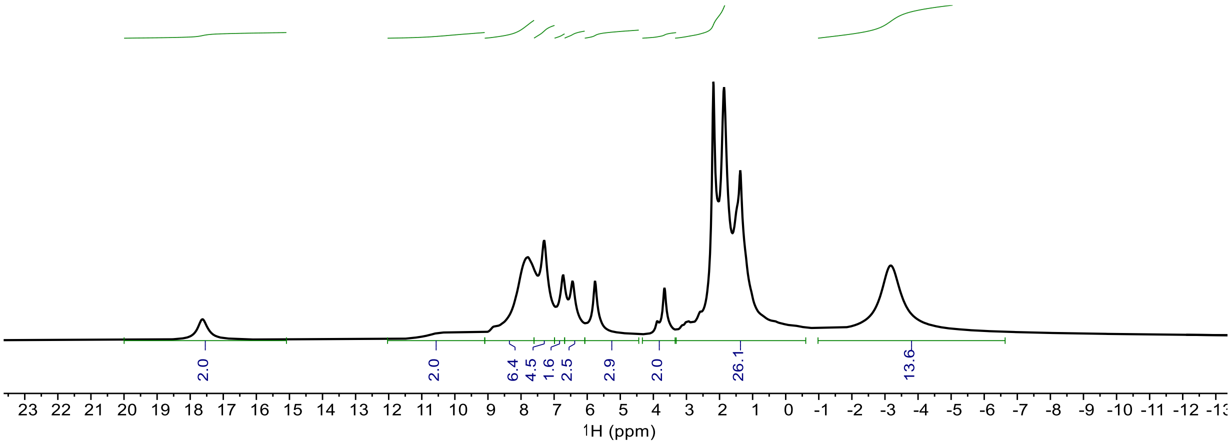

Figure S4. ^{1}H NMR (400 MHz) spectrum K[**1**] without the addition of 12-crown-4 in MeCN-d_3 at 298 K.

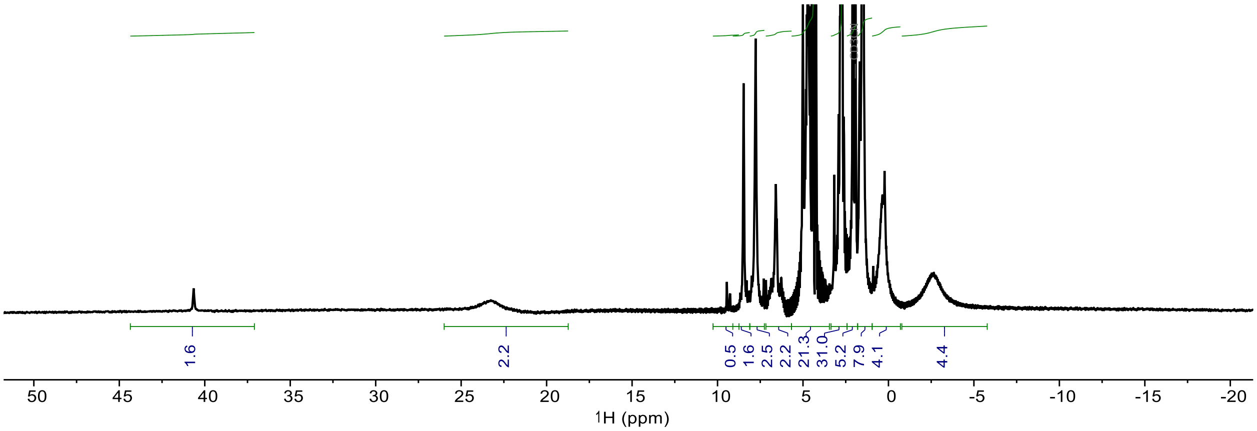

Figure S5. ^{1}H NMR (400 MHz) spectrum of crystals of [K(12-crown-4)$_n$][**2**] (n = 1–2) in MeCN-d_3 at 298 K.

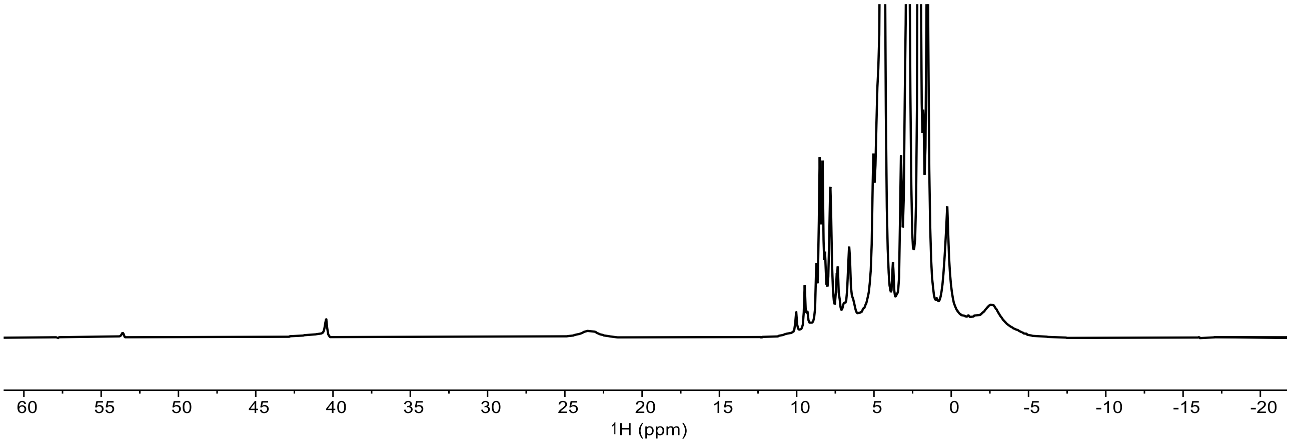

Figure S6. ^{1}H NMR (400 MHz) spectrum of crude reaction containing [K(12-crown-4)$_n$][**2**] (n = 1–2) in MeCN-d_3 at 298 K.

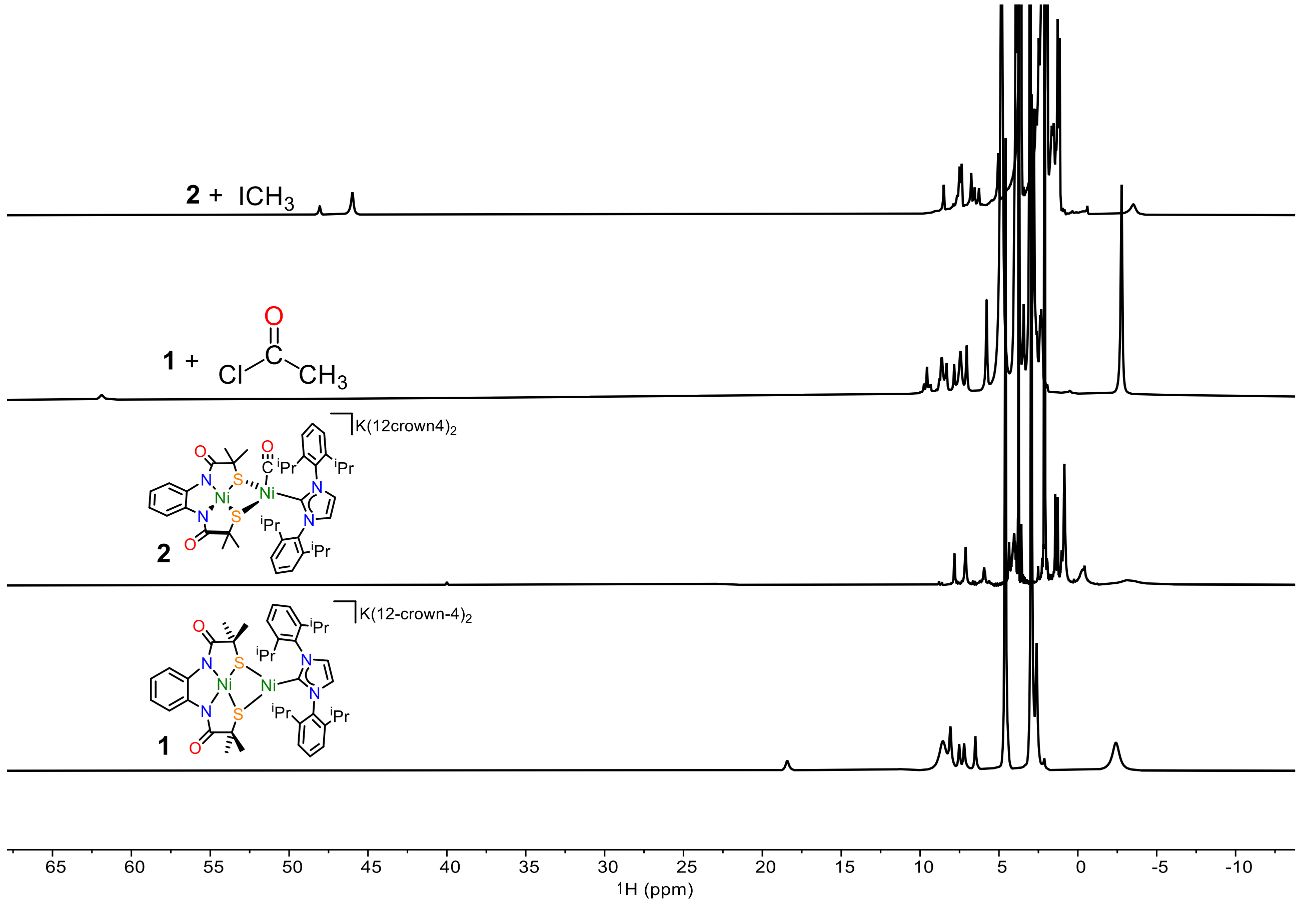

Figure S7. ^{1}H NMR (400 MHz) spectra of reactions attempting to generate nickel-acyl species in MeCN-d_3 at 298 K.

5.0. FTIR spectra of metal complexes

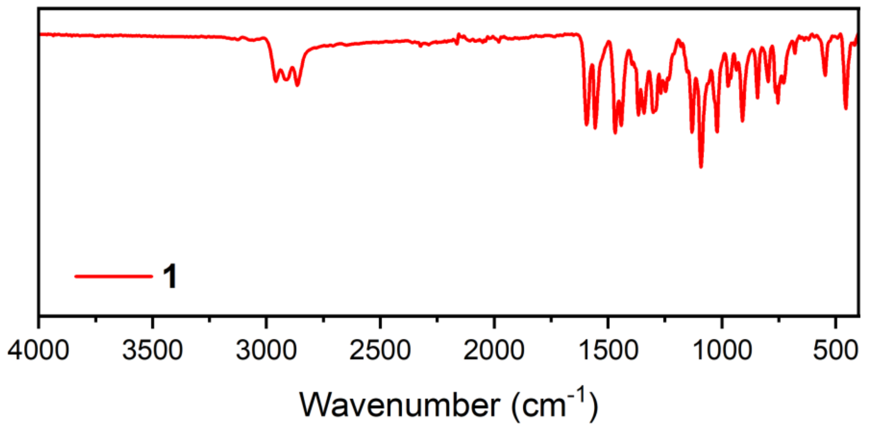

Figure S8. FTIR spectrum of [K(12-crown-4)$_2$][**1**] (solid).

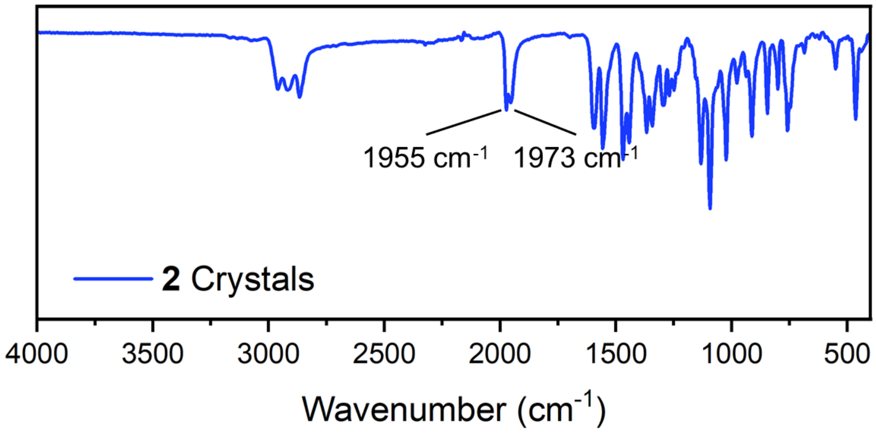

Figure S9. FTIR spectrum of [K(12-crown-4)$_n$][**2**] (solid), a mixture of 12-crown-4 salts (n = 1–2).

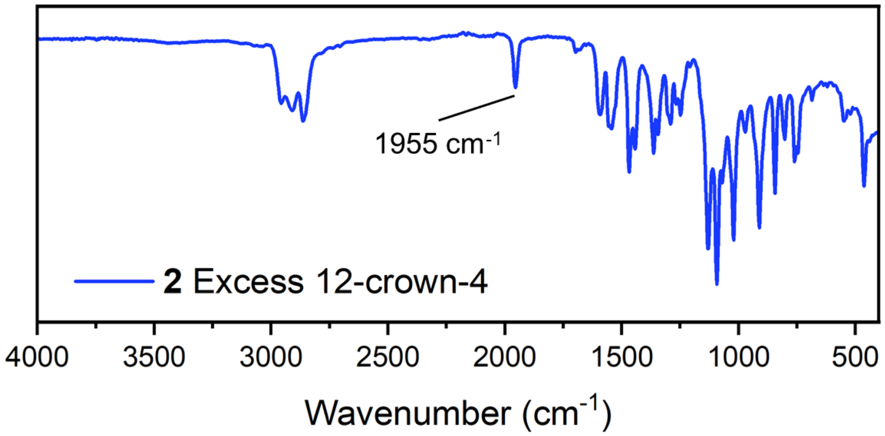

Figure S10. FTIR spectrum of [K(12-crown-4)$_2$][**2**] (solid) prepared by addition of excess 12-crown-4.

Table S1. Comparison of **2** to previously reported, structurally authenticated Ni^{1+}–CO complexes.

**Complex**	**Reference**	**vCO (cm^{-1})**	**Ni–C (Å)**	**C–O (Å)**	**Ni–C–O (°)**
$[(N(CH_2CH_2SBu^t)_3)NiCO]^+$	6	2026	1.85(1)	1.15(1)	177.5(9)
$(PhB(CH_2S^tBu)_3)NiCO$	7	1999	1.755(8)	1.127(11)	170.9(7)
$(PhB(CH_2S^tAd)_3)NiCO$	8	1997	1.815(4)		175.0
(4,5-bis(diisopropylphosphino)-2,7,9,9-tetramethyl-9H-acridin-10-ide)NiCO	9	1936	1.765(1)	1.149(2)	174.4(1)
[K(12-crown-4)$_2$][**2**]	This work	1955	N/A	N/A	N/A
[K(12-crown-4)$_1$][**2**]	This work	1973	1.793(4)	1.096(5)	178.6(4)

6.0. UV-visible absorption spectra

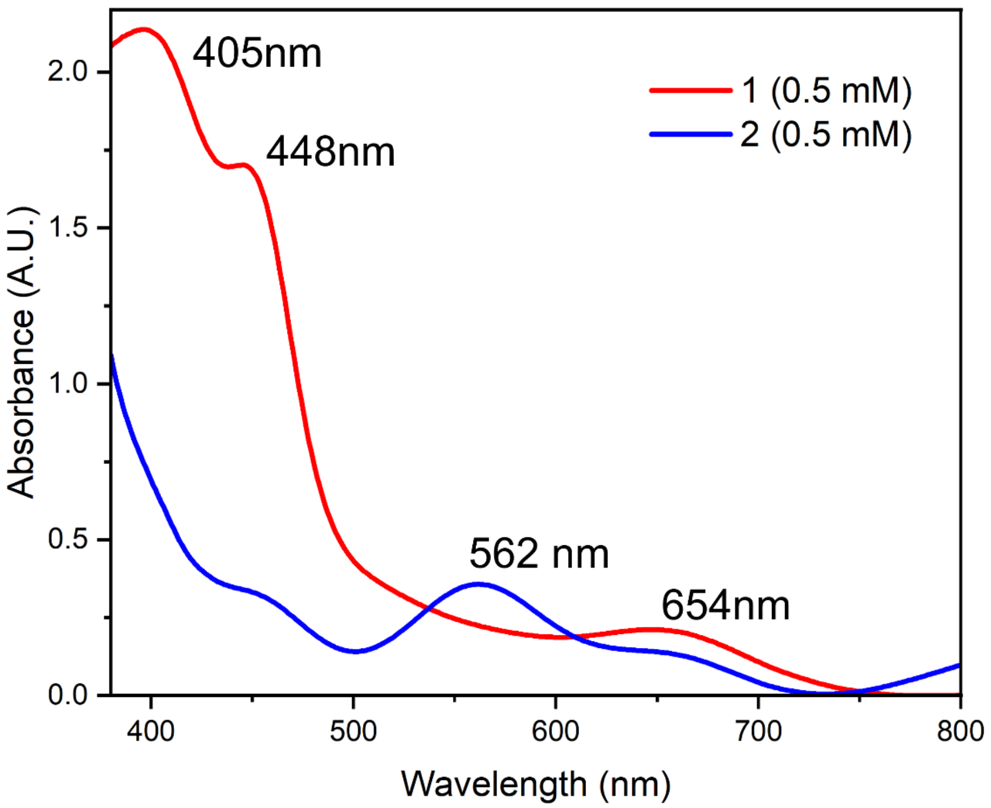

Figure S11. UV-vis spectra of [K(12-crown-4)$_2$][**1**] (red) and [K(12-crown-4)$_2$][**2**] (blue) as 0.5 mM solutions in MeCN.

7.0. Electrochemistry

The electrochemistry of compounds [**1**] (3.9 mM) and [**2**] (2.4 mM) were investigated in MeCN solution with 0.2 M [N(^{t}Bu)$_4$][PF_6] as the supporting electrolyte with glassy carbon working and counter electrodes. Measurements were taken in a nitrogen glove box to preclude interference from atmospheric oxygen and moisture. $CoCp_2$ was used as an internal reference throughout, as the Fc/Fc$^+$ redox couple was masked by irreversible oxidation peaks, and these values have been subsequently translated relative to the Fc/Fc$^+$ taking the value of $CoCp_2$/$CoCp_2^+$ as –1.35 V vs Fc/Fc$^+$ in MeCN[10] to simplify comparisons to other data.

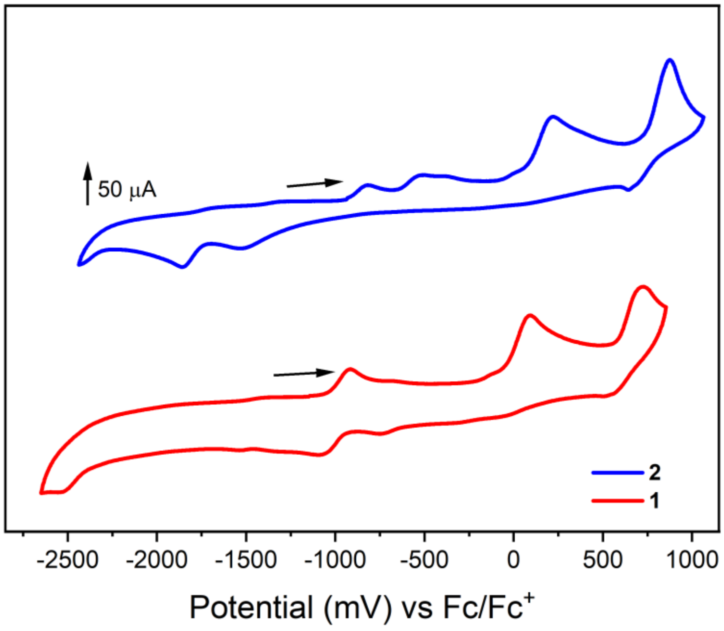

Figure S12. Cyclic voltammograms for [**1**]$^–$ (3.9 mM) and [**2**]$^–$ (2.4 mM) in MeCN with 0.2 M [N(^{t}Bu)$_4$][PF_6] as the supporting electrolyte with glassy carbon working and counter electrodes. $CoCp_2$ was used as an internal reference, and values are subsequently reported vs Fc/Fc$^+$ taking the value of $CoCp_2$/$CoCp_2^+$ as –1.35 V vs Fc/Fc$^+$ in MeCN.[10]

The cyclic voltammograms for both complexes [**1**]$^–$ and [**2**]$^–$ exhibit irreversible oxidations at 90 and 220 mV, and 725 and 870 mV for [**1**]$^–$ and [**2**]$^–$ respectively. The origin of these signals is not fully understood but it is speculated these could be the result of ligand-based oxidation processes. The shape of each signal is similar between both complexes, although they appear shifted anodically in [**2**]$^–$, possibly due to the electron-withdrawing effect of the additional coordinated CO. The irreversibility is likely a consequence of sample decomposition, and repeated cycles read to the gradual disappearance of all signals in the voltammogram. Polishing of the electrodes leads to recovery of the initial voltammogram. Also note that the reversible Ni^{1+}/Ni^{2+} redox couple at –842 mV reported in the main text (Figure 5) becomes irreversible upon sweeping higher potentials – it is thought that the oxidation at –520 mV leads to a

structural change that renders the Ni^{1+}/Ni^{2+} couple irreversible. A consequence of CO coordination in [**2**]$^{-}$ is the appearance of irreversible reduction events between –1550 and –1857 mV (Figure S12, which are absent in the electrochemistry of [**1**]$^{-}$). These reductive events may be attributed to formation of Ni^{0}, and Ni^{0}/Ni^{1+} redox couples in NHC stabilised complexes in this region have previously been previously in the literature.[11,12] This effect is attributed to the π-accepting nature of the bound CO in [**2**]$^{-}$, which stabilises Ni^{0} (relative to [**1**]$^{-}$) making these redox transformations more accessible. The irreversibility is likely due to sample decomposition.

Scan rate dependence studies were completed for the Ni^{1+}/Ni^{2+} redox couple for [**1**]$^{-}$ and [**2**]$^{-}$, indicating full chemical reversibility for compound [**1**]$^{-}$. Loss of signal resolution was observed upon increasing the scan rate for compound [**2**]$^{-}$.

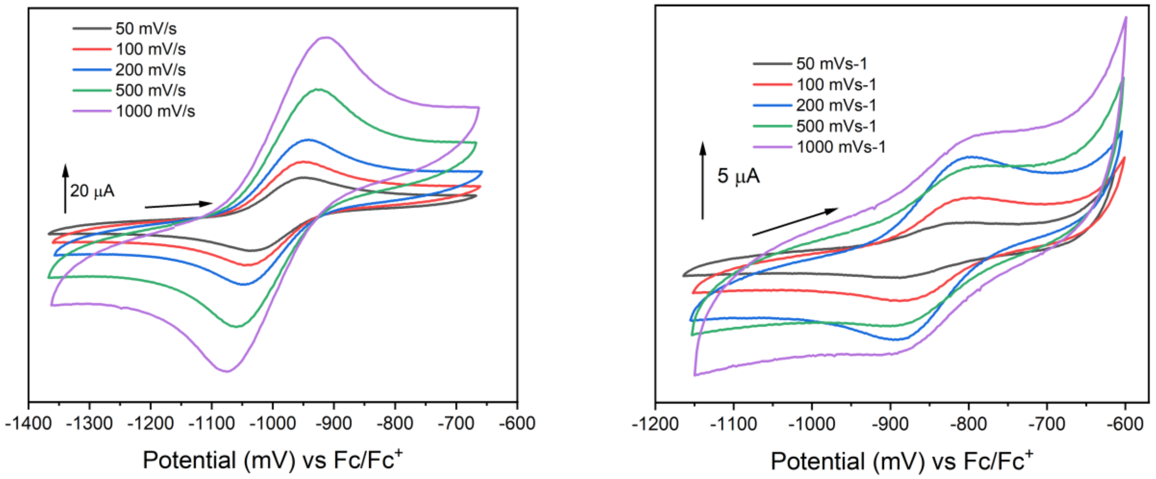

Figure S13. Left: Scan rate dependence for Ni^{1+}/Ni^{2+} for compound [**1**]$^{-}$. Right: Scan rate dependence for Ni^{1+}/Ni^{2+} for compound [**2**]$^{-}$.

Table S2. Selected Ni^{1+}/Ni^{2+} couples for well-defined 3-coordinate Ni complexes. *Converted from NHE reference $FeCp_2/FeCp_2^+$ taken as + 690 mV vs NHE (in MeCN).[13] These comparisons must be considered an approximation owing to differences incurred by solvent, electrolyte concentration, temperature *etc*. (^{t}BuXantphos) = 9,9-Dimethyl-4,5-bis(di-tert-butylphosphino)xanthene, IMes = 1,3-Bis(2,4,6-trimethylphenyl)-1,3-dihydro-2H-imidazol-2-ylidene dtbpe = 1,2-bis(di-tert-butylphosphino)ethane, TIMEN = tris[2-(3-tert-butylimidazol-2-ylidene)ethyl]amine, IPr = 1,3-di(2,6-di-isopropylphenyl)imidazolin-2-ylidene), Ar = 2,6-di-isopropylphenyl).

**Compound**	**Ni^I/Ni^{2+} redox potential (V vs Fc/Fc$^+$)**	**Solvent**	**Electrolyte (Concentration/ M)**	**Reference**
(^{t}BuXantphos)Ni(*o*-Tol)	–1.51	THF	$[N(^nBu)_4][PF_6]$ (0.4)	[14]
(IMes)(Cp)NiCl	–1.30*	MeCN	$[N(^nBu)_4][BF_4]$ (0.1)	[13]
(dt bpe)Ni(CH_2CMe_3)	–1.25	THF	$[N(^nBu)_4][PF_6]$ (0.3–0.5)	[15]
Ni(TIMEN^{t}Bu)	–1.09	THF	$[N(^nBu)_4](ClO_4)$ (0.1)	[16]
**[1]**$^–$	–0.99	MeCN	$[N(^nBu)_4][PF_6]$ (0.2)	This work
(dtbpe)Ni(NH(2,6-($CHMe_2)_2C_6H_3$))	–0.90	THF	$[N(^nBu)_4][PF_6]$ (0.3–0.4)	[17]
**[2]**$^–$	–0.84	MeCN	$[N(^nBu)_4][PF_6]$ (0.2)	This work
(IPr)Ni(NHAr)	– 0.84	THF	$[N(^nBu)_4][PF_6]$ (0.3–0.4)	[18]

8.0. EPR Spectroscopy

The CW-EPR spectrum of a 3 mM solution of [**1**]$^{-}$ in 2-methyltetrahydrofuran was recorded at a temperature of 93 K on a Magnettech ESR5000 benchtop spectrometer (Bruker) equipped with a TCH04 N_2 variable temperature unit. The sample was loaded in a 4 mm OD, 2 mm ID quartz tube sealed with a J Young valve. The measurements were performed using a microwave power of 10 mW, a field modulation amplitude of 0.5 mT at 100 kHz and a magnetic field sweep rate of 2 mTs^{-1}. A measurement of the CW-EPR spectrum of [**1**]$^{-}$ at room temperature was attempted but did not give any observable signal.

The CW-EPR of a 5 mM solution of [**2**]$^{-}$ in 2-methyltetrahydrofuran was recorded at a temperature of 25 K on a Bruker Elexsys E580 spectrometer (Bruker) equipped with an ER 4118X-MD5 dielectric ring resonator and a closed-circuit Helium cryostat (Cryogenic Ltd.). The sample was loaded in the cryostat as a 4 mm OD, 3 mm ID clear fused quartz tube (Wilmad 707-SQ-250M) flame-sealed under vacuum. Measurements shown in the main manuscript were performed using a microwave power of 15 µW, a field modulation amplitude of 0.2 mT at 100 kHz and a magnetic field sweep rate of 1.3 mTs^{-1}. Power-dependent measurements were carried out to as shown in **Figure S13**.

For both spectrometers the magnetic field was calibrated using a DPPH standard (g = 2.0036).[19]

Simulations of the CW-EPR spectra were performed using the `pepper` function of the EasySpin 5.2.36 toolbox[20] running on MATLAB R2021a. The magnetic parameters were refined using the Nelder-Mead simplex algorithm as implemented in the `esfit` function from the same toolbox.

The displayed simulations are based on the following magnetic parameters:

Sample	*g* values			*g* strain			Anisotropic residual linewidth		
[**1**]$^{-}$	2.0618	2.0706	2.5379	0.026	0.083	0.136	1.8 MHz	4.1 MHz	3.5 MHz
[**2**]$^{-}$	2.2674	2.1136	1.9972	0.046	0.014	0.004	6.0 MHz	7.3 MHz	7.6 MHz

The anisotropic residual linewidth was introduced as `HStrain` and is given as the full width at half height.

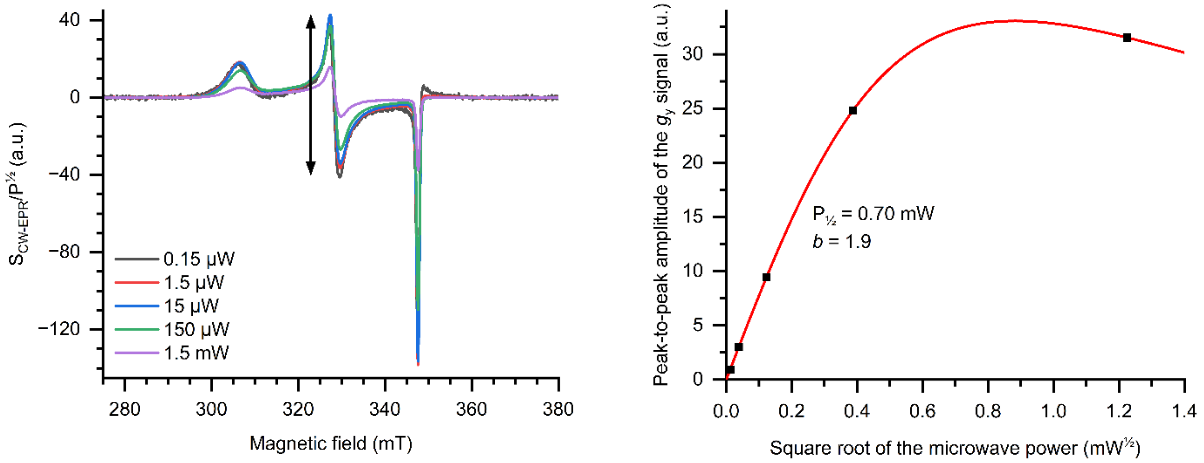

Figure S14. Power-dependent spectra of [**2**]⁻ recorded at 25 K with a field modulation amplitude of 5.0 G at 100 kHz. The peak-to-peak amplitude of the g_y component was analysed according to the equation $S = \frac{A \cdot \sqrt{P}}{(1+P/P_{½})^{b/2}}$;[21] the $P_{½}$ and b values are reported in the figure.

Table S3. Tabulated EPR parameters

Compound	g_1	g_2	g_3	g_{iso}	reference
**[1]$_{exp}$**	2.538	2.071	2.062	2.224	This work
**[1]$^{-Y}_{DFT}$**	2.212	2.115	2.071	2.133	This work
**[1]$^{-T}_{DFT}$**	2.29	2.219	2.065	2.191	This work
**[1]$^{-T}_{CASSCF/NEVPT2}$**	2.862	2.218	2.085	2.388	This work
**[2]$^{-}_{exp}$**	2.267	2.114	1.997	2.126	This work
**[2]$^{-}_{DFT}$**	2.242	2.148	2.018	2.136	This work
**nacnacNiCO**	2.166	2.193	2.014	2.124	[22]
**AzurinNiCO**	2.283	2.199	2.006	2.163	[23]
**ACS A$_{NiFeC}$**	2.067	2.026		2.047	[24,25]
**ACS A$_{red}$**	2.56	2.10		2.223	[26]

9.0. <u>Crystallographic data</u>

Crystal data and structure refinement for [K(12-crown-4)$_2$][**1**].

Empirical formula	$C_{57}H_{84}KN_4Ni_2O_{10}S_2$
Formula weight	1205.92
Temperature/K	153(7)
Crystal system	triclinic
Space group	P-1
a/Å	13.26260(10)
b/Å	14.02110(10)
c/Å	19.5456(2)
α/°	105.5120(10)
β/°	96.4910(10)
γ/°	104.4310(10)
Volume/Å^3	3328.25(5)
Z	2
ρ_{calc}g/cm^3	1.203
μ/mm^{-1}	2.270
F(000)	1282.0
Crystal size/mm^3	0.1 × 0.1 × 0.1
Radiation	Cu Kα (λ = 1.54184)
2Θ range for data collection/°	6.84 to 159.242
Index ranges	$-16 \le h \le 16$, $-17 \le k \le 16$, $-20 \le l \le 24$
Reflections collected	42626
Independent reflections	13737 [R_{int} = 0.0374, R_{sigma} = 0.0353]
Data/restraints/parameters	13737/636/879
Goodness-of-fit on F^2	1.055
Final R indexes [I>=2σ (I)]	R_1 = 0.0374, wR_2 = 0.1029
Final R indexes [all data]	R_1 = 0.0428, wR_2 = 0.1065
Largest diff. peak/hole / e Å^{-3}	0.42/–0.38

Crystal data and structure refinement for [K(12-crown-4)$_1$][**2**].

Empirical formula	$C_{52}H_{71}KN_5Ni_2O_7S_2$
Formula weight	1098.77
Temperature/K	150(2)
Crystal system	monoclinic
Space group	$P2_1/c$
a/Å	22.0579(4)
b/Å	13.9951(2)
c/Å	19.8133(3)
α/°	90
β/°	105.3505(17)
γ/°	90
Volume/Å^3	5898.22(17)
Z	4
ρ_{calc}g/cm^3	1.237
μ/mm^{-1}	2.480
F(000)	2324.0
Crystal size/mm^3	0.165 × 0.112 × 0.062
Radiation	CuKα (λ = 1.54184)
2Θ range for data collection/°	7.562 to 158.836
Index ranges	$-28 \leq h \leq 27$, $-17 \leq k \leq 16$, $-23 \leq l \leq 25$
Reflections collected	45202
Independent reflections	12152 [R_{int} = 0.0481, R_{sigma} = 0.0447]
Data/restraints/parameters	12152/0/617
Goodness-of-fit on F^2	1.050
Final R indexes [I>=2σ (I)]	R_1 = 0.0503, wR_2 = 0.1392
Final R indexes [all data]	R_1 = 0.0750, wR_2 = 0.1537
Largest diff. peak/hole / e Å^{-3}	0.74/–0.29

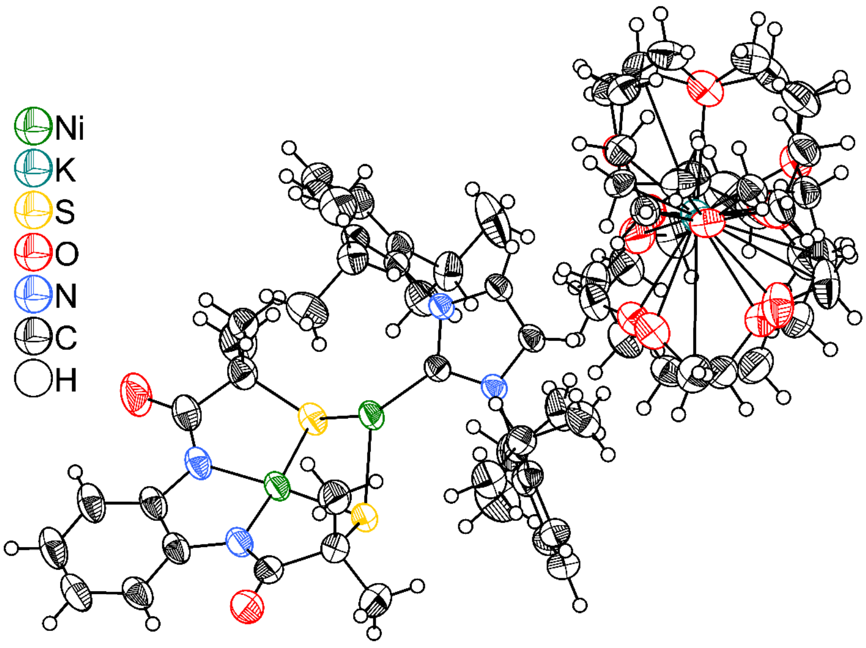

Figure S15. Single crystal X-ray structure of $[K(12\text{-crown-}4)_2][\mathbf{1}]$. 12-crown-4 ligands disordered over two positions. Anisotropic displacement ellipsoids depicted at 50% probability and hydrogen atoms are pictured as spheres of arbitrary radii.

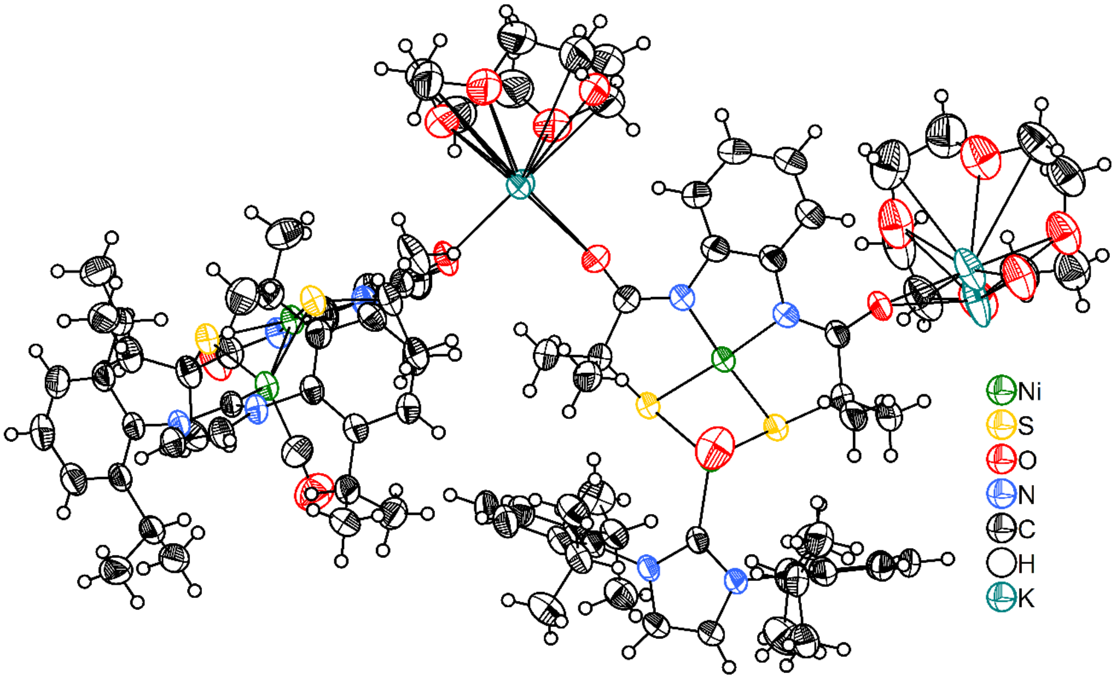

Figure S16. Single crystal X-ray structure of $[K(12\text{-crown-}4)_1][\mathbf{2}]$ showing polymeric structure in the solid state. The potassium ion is disordered between two positions, and for clarity the position with the highest relative occupancy is displayed (80%). Anisotropic displacement ellipsoids depicted at 50% probability and hydrogen atoms are pictured as spheres of arbitrary radii.

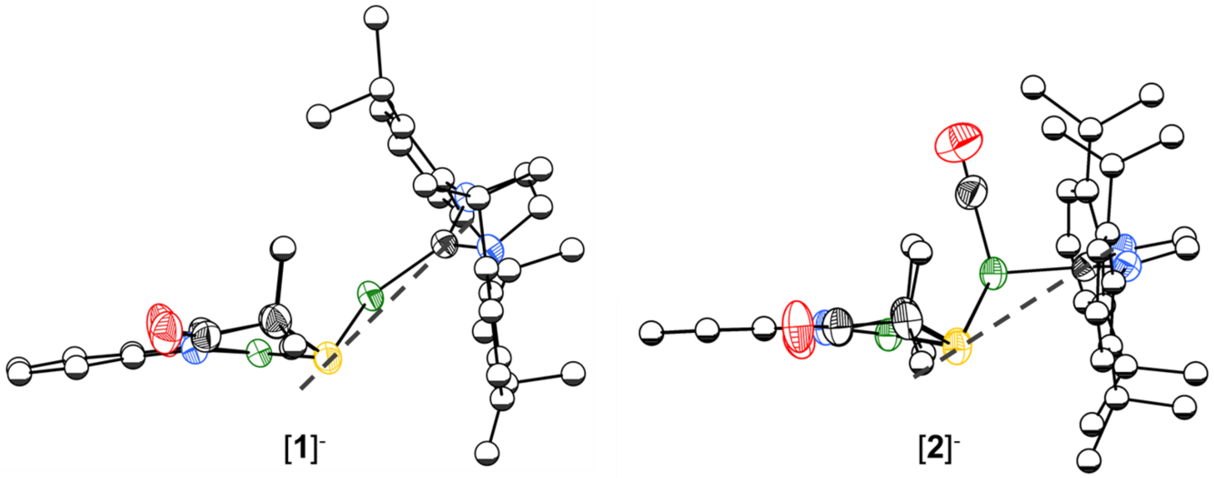

Figure S17. Single crystal X-ray structure of the anion [**1**] (left) and [**2**] (right) showing "butterfly" type arrangement of N_2S_2 ligand about the Ni2 site. Distance between S1–S2–C1 plane and Ni1 0.3037(7) Å ([**1**]) and 0.9163(14) Å ([**2**]). Anisotropic displacement ellipsoids depicted at 50% probability and hydrogen atoms are omitted.

10.0. Computational Details

Geometry optimizations and frequency calculations were performed using the Gaussian program package (versions 09 and 16). Optimized geometries were calculated from the anionic portion of crystal structure coordinates, omitting potassium and associated 12-crown-4 molecules, using the B3LYP functional and the scalar relativistically recontracted version of the Aldrichs triple-z basis set (def2-TZVP).[27–29] Grimmes dispersion correction (GD3) was used on all atoms. Energy minima were confirmed by the presence of no imaginary frequencies in the vibrational calculation. Relaxed surface scans were performed with BP86 functional and def2-SVP basis set due to computational constraints. Transition states were assessed by the presence of a single imaginary frequency productive to the reaction coordinate. After identification of minima and transition states, single point calculations were performed with B3LYP/def2-TZVP/GD3 with MeCN as the implicit solvent, and the relative free energy values are shown in Table S3. Cations were omitted from the calculations in all cases.

The calculated IR stretching frequency for $[\mathbf{2}]^{-}$ was ν_{co} = 2037 cm^{-1}. After application of the scaling factor associated with the methodology (0.965) results in a ν_{COcalc} = 1966 cm^{-1} in agreement with the experimental value of 1955 cm^{-1}.

Table S4. Comparison of key bond metrics to crystal data. SP = square planar, NHC = N-heterocyclic carbene.

	[**1**]$^{-}$		[**2**]$^{-}$	
	Exp (Å)	Opt (Å)	Exp (Å)	Opt (Å)
**Ni···Ni$_{SP}$**	2.6303(4)	2.586	2.5820(6)	2.754
**Ni1–C$_{NHC}$**	1.9210(15)	1.916	1.977(3)	2.003
**Ni1–S**	2.2636(6), 2.2689(5)	2.329, 2.359	2.3331(7), 2.3403(9)	2.447
**Ni1–CO**	-	-	1.792(4)	1.810
**S–Ni–C$_{NHC}$**	139.25(5), 122.49(5)	156.0, 114.2	126.17(11), 121.36(11)	110.5
**τ_4(Ni)**	-	-	0.793	0.786

Example input files for Gaussian16:

Optimization (with frequency and NBO calculation):

```
%nprocshared=16
%mem=47GB
%chk=DW020_B3LYP_NBO.chk
# opt freq=noraman ub3lyp Def2TZVP empiricaldispersion=gd3 pop=(full,nboread) SCRF(Solvent=Acetonitrile)

CO TZVP

-1 2
 {atom coorindates}

$NBO PLOT $END
```

Relaxed scan:

```
%nprocshared=16
%mem=47GB
%chk=CO_Scan.chk
# opt(modredundant) BP86/def2SVP

scan CO

-1 2
 Ni                  -0.29969700    0.32951000   -0.63122700
 {atom coordinates}

 B 1 104 S 50 -0.1
```

TDDFT:

```
%nprocshared=16
%mem=47GB
%chk=DW020_TDDFT_20.chk
# ub3lyp Def2TZVP empiricaldispersion=gd3 SCRF(Solvent=Acetonitrile) td=(nstates=20)

CO TZVP

-1 2
{atom coordinates}
```

10.1. Thermodynamics from DFT calculations

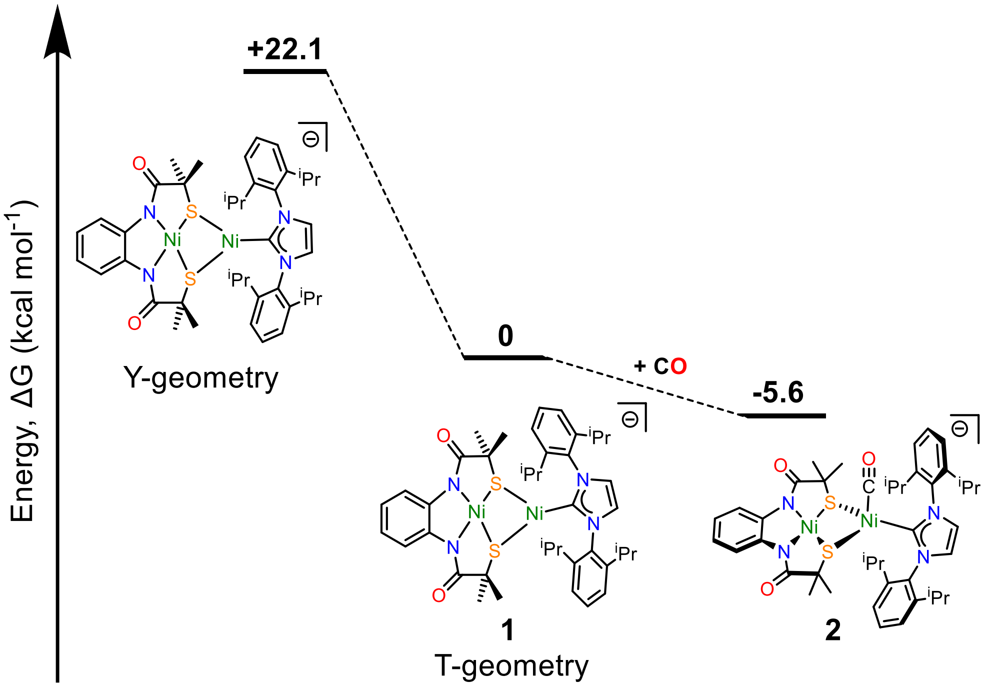

Figure S18. Relative free energy of optimized structures [**1**]$^-$ and [**2**]$^-$, and enforced Y-geometry of [**1**]$^-$.

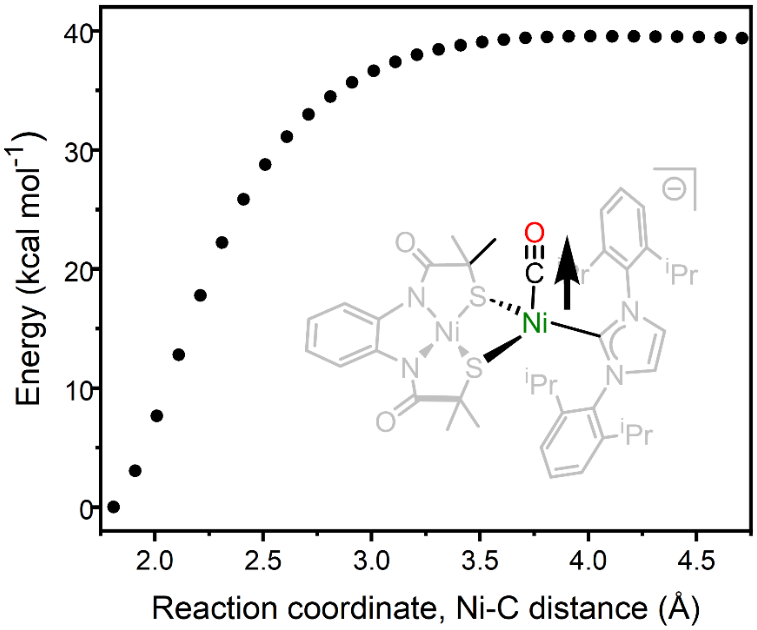

Figure S19. Relaxed scan (BP86/def2-SVP) increasing the Ni–C bond distance, illustrating barrierless transition upon loss of CO.

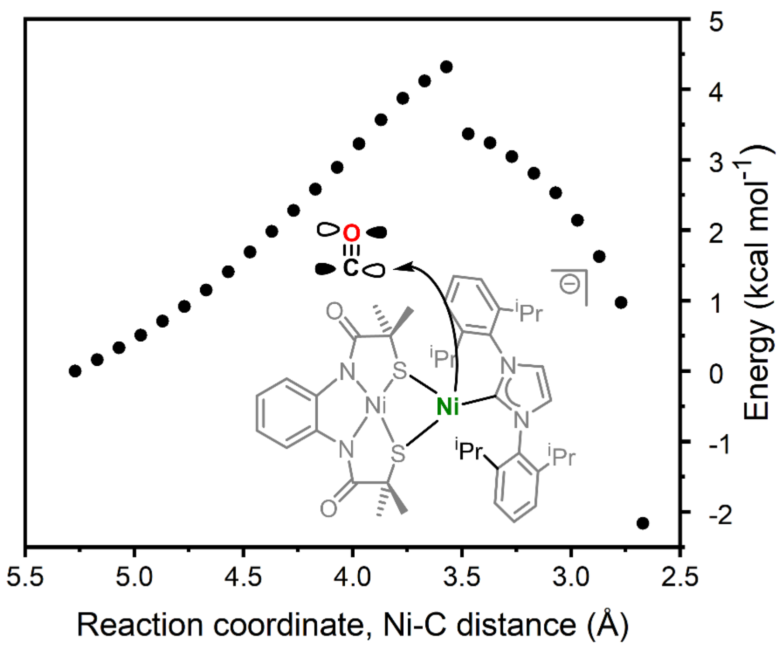

Figure S20. Example relaxed scan (BP86/def2-SVP) of decreasing Ni–C bond distance, sharp decrease in energy around 3.5 Å is due to NHC ligand rotation. Both maxima were optimized as transition states, and while had only 1 imaginary frequency, both had intensities of <91 cm^{-1} indicating they are not true transition states.

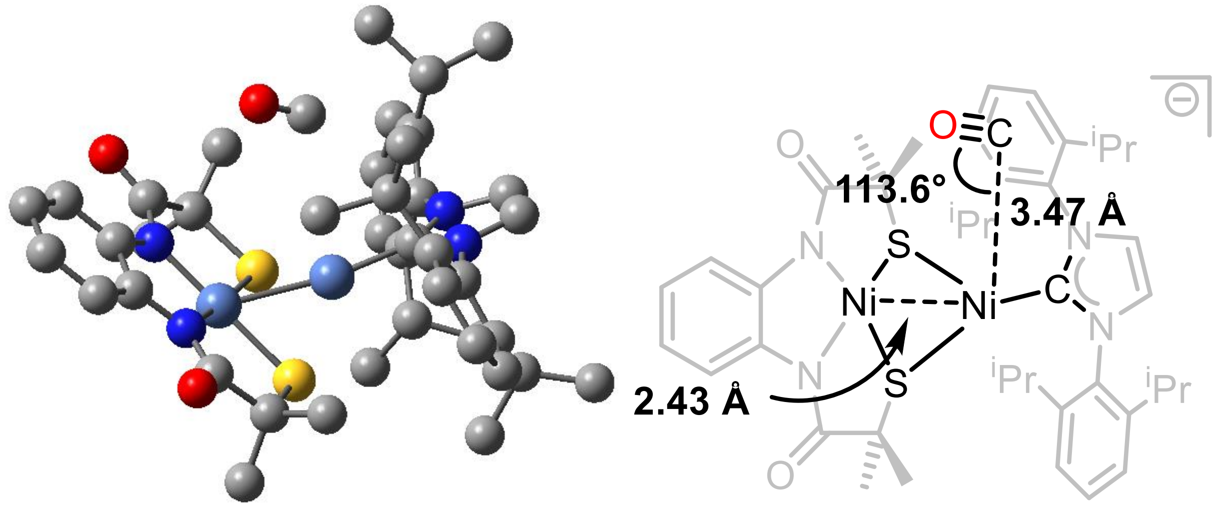

Figure S21. Pseudo-transition state from above scan showing angle of approach by CO.

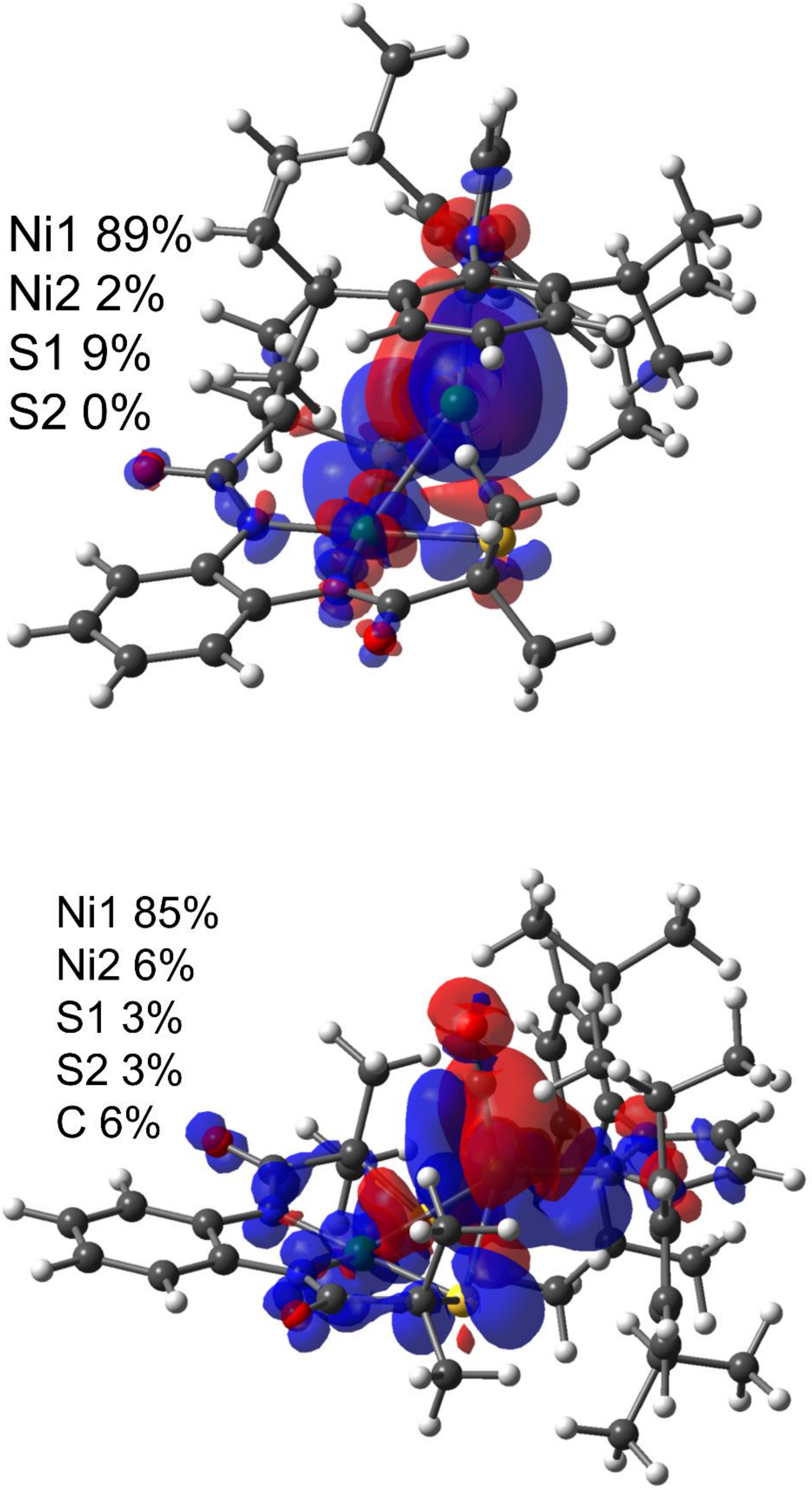

Figure S22. Spin density plots for [**1**]$^{-}$ (top) and [**2**]$^{-}$ (bottom) displayed at isodensity value 0.008 au. Numbers indicate spin density values at atoms surrounding nickel.

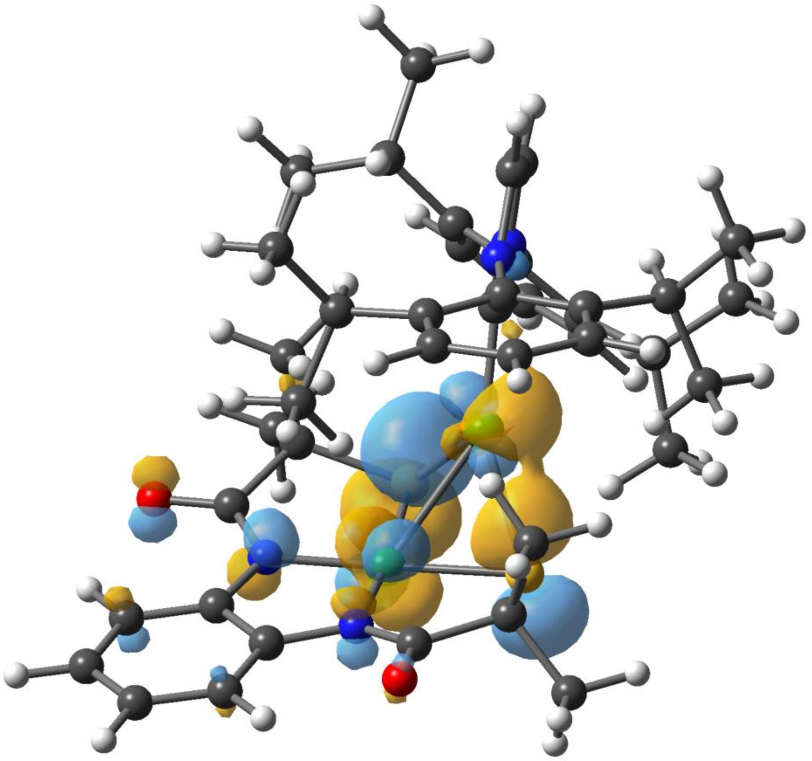

Figure S23. SOMO–1 orbital of [**1**]$^{-}$, displaying π-interaction between three-coordinate nickel and sulfur which allows metal-to-ligand transfer of charge density. Isodensity value 0.002 au.

10.2. Time-dependent density functional theory (TDDFT)

TDDFT calculations were performed with on the optimized structure of [**1**]$^{-}$ and [**2**]$^{-}$ with B3LYP/def2-TZVP/gd3 in acetonitrile as solvent, using 20 roots. The visible region of the spectrum was well reproduced for both complexes. For the optimized structure of [**2**]$^{-}$, the excited state 9 (564 nm) reproduces the absorbance at 562 nm well and is comprised of 12 transitions.

Despite decades of research into the A-cluster, there is little discussion in the literature of the role of the Ni_D site, other than discounting it participating in bonding with any substrates. However, we can extrapolate from discussions of other metallocofactors that it may tune the redox potential of the Ni_P site,[30] form metal-metal bonds to store electrons,[31] or provide rigidity in the coordination environment of Ni_P.[32] While our electrochemical and computational studies reveal no evidence for nickel-nickel bonding, TDDFT analysis suggests the square planar nickel facilitates charge transfer to the π*-antibonding orbital of CO, which may activate the CO for subsequent functionalization. While the energy of the absorbance in [**2**] (2.2 eV) is likely too large to be useful in biology, studies on ligand modification in platinum complexes used in artificial photosynthesis have found the absorbance energy can be tuned by ~1 eV through modification of the ligand.[33] The difference in ligand identities between [**2**] and the A-cluster may result in a transition energy accessible in biological systems.

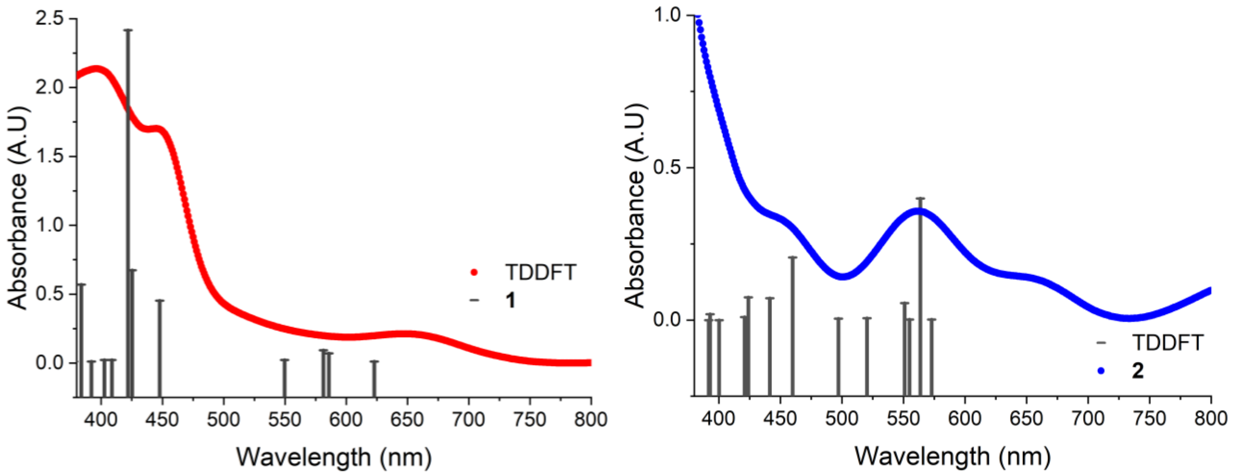

Figure S24. Experimental UVvis spectra for [**1**]$^{-}$ and [**2**]$^{-}$ (solid lines) overlayed with calculated transitions (grey bars) from TDDFT calculations.

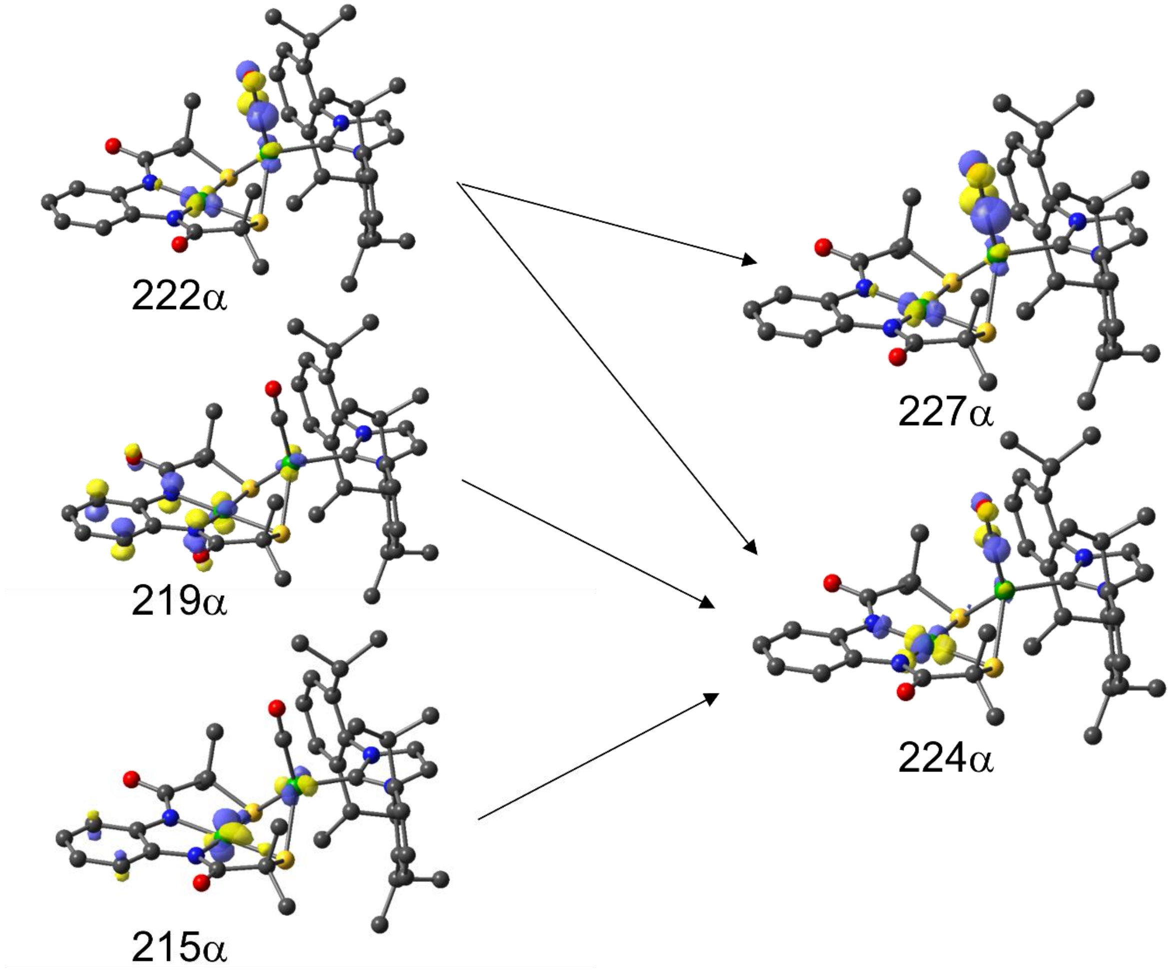

Figure S25. Graphical representation of the α-orbital transitions in [**2**]$^{-}$ contributing to excited state 9 in the TDDFT calculation (B3LYP/def2-TZVP/gd3/MeCN).

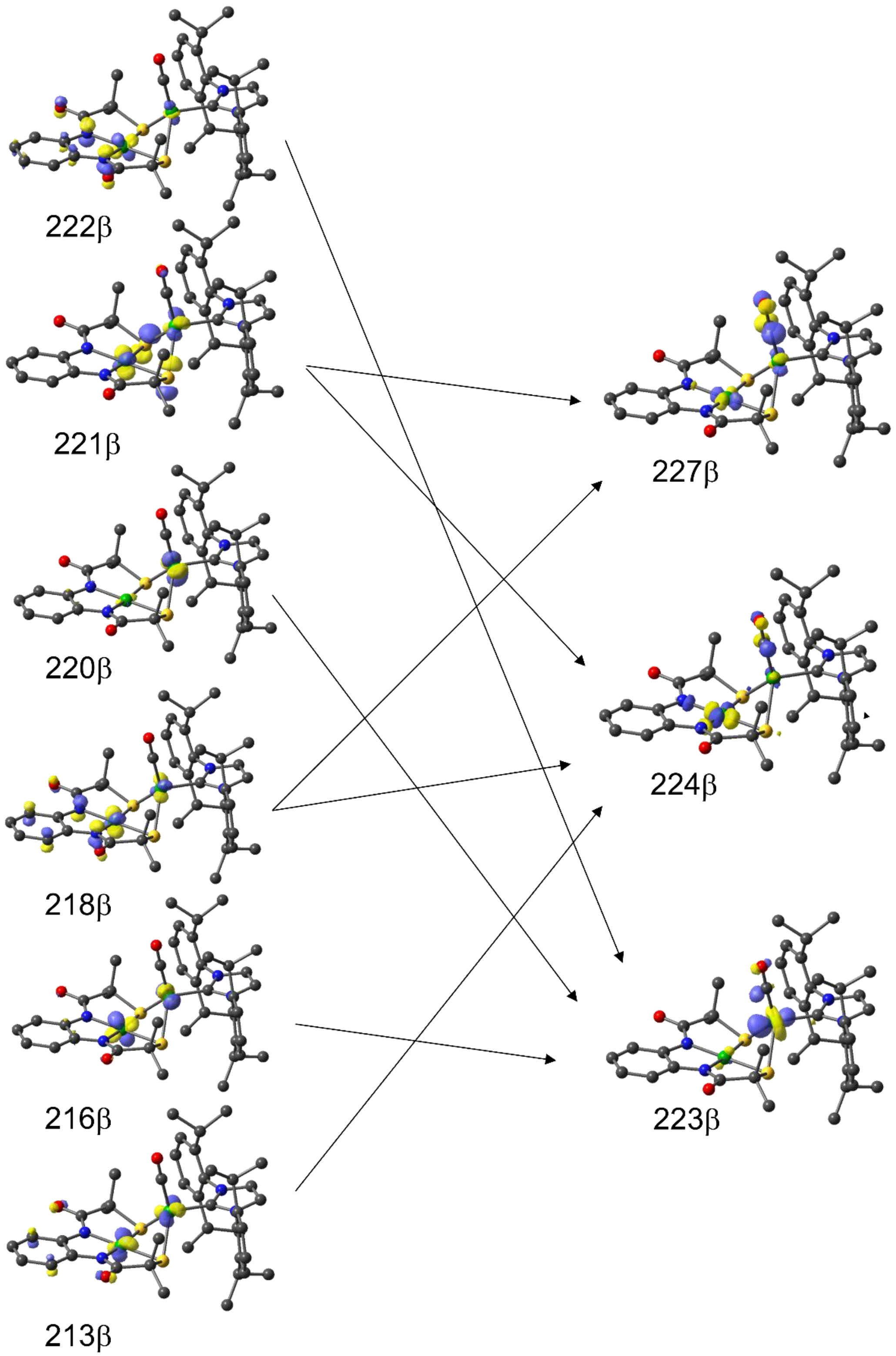

Figure S26. Graphical representation of the β-orbital transitions in [**2**]$^{-}$ contributing to excited state 9 in the TDDFT calculation (B3LYP/def2-TZVP/gd3/MeCN).

Extended output from TDDFT calculations

Excited State 6: 2.630-A 1.2043 eV 1029.49 nm f=0.0000 <S**2>=1.479

218A -> 224A 0.18212

218A -> 227A -0.13526

221A -> 224A -0.24732

221A -> 227A 0.14409

223A -> 224A 0.17008

204B -> 223B 0.19459

211B -> 223B -0.16562

212B -> 223B 0.41059

215B -> 223B 0.47320

215B -> 234B 0.10736

218B -> 223B 0.19672

219B -> 224B -0.31709

219B -> 227B 0.21508

221B -> 223B -0.22555

222B -> 224B -0.11292

Excited State 7: 2.018-A 1.4346 eV 864.25 nm f=0.0011 <S**2>=0.768

196B -> 223B 0.10903

202B -> 223B 0.35759

202B -> 234B 0.10458

205B -> 223B 0.18492

206B -> 223B -0.24125

208B -> 223B 0.13620

214B -> 223B 0.76158

214B -> 234B 0.18437

217B -> 223B 0.11830

219B -> 223B 0.16522

Excited State 8: 2.199-A 2.1643 eV 572.87 nm f=0.0001 <S**2>=0.959

206A -> 224A	-0.15341
206A -> 227A	0.11325
217A -> 224A	-0.17257
217A -> 227A	0.11610
220A -> 224A	0.20117
220A -> 227A	-0.13526
223A -> 224A	0.47090
223A -> 227A	-0.27893
206B -> 224B	0.13848
216B -> 224B	0.24674
216B -> 227B	-0.16526
217B -> 224B	0.16384
217B -> 227B	-0.10391
222B -> 224B	0.48163
222B -> 227B	-0.27350

Excited State 9: 2.115-A 2.1996 eV 563.66 nm f=0.0245 <S**2>=0.868

215A -> 224A	-0.12205
219A -> 224A	0.11560
222A -> 224A	0.25120
222A -> 227A	-0.17374
213B -> 224B	-0.10234
216B -> 223B	0.30201
218B -> 224B	0.14090
218B -> 227B	-0.10256
220B -> 223B	-0.18168
221B -> 224B	0.24196
221B -> 227B	-0.14036
222B -> 223B	0.75035

Excited State 10: 3.326-A   2.2347 eV 554.80 nm f=0.0001 <S**2>=2.516

206A -> 224A	0.44445
206A -> 227A	-0.31550
217A -> 224A	-0.14483
218A -> 224A	0.14716
221A -> 224A	0.23139
221A -> 227A	-0.15412
223A -> 224A	0.11947
203B -> 224B	-0.11074
205B -> 224B	-0.36824
205B -> 227B	0.25384
206B -> 224B	-0.22047
206B -> 227B	0.15177
216B -> 224B	0.13302
217B -> 224B	-0.16600
217B -> 227B	0.10771
220B -> 224B	0.18924
220B -> 227B	-0.10616
222B -> 224B	0.15058

10.3. EPR Calculations

EPR parameters were calculated using both DFT and multireference CASSCF methods in ORCA version 5.0.3.[34] For the DFT calculations, in accordance with a previous report showing that the hybrid functional B3LYP was able to reproduce the EPR parameters of a tetrahedral nickel CO adduct,[35] we used B3LYP in combination with the EPR-optimized basis set CP(PPP) on both Ni centers, ZORA-def2-QZVPP on all atoms directly bound to Ni, and ZORA-def2-SVP on all other atoms. The auxiliary basis set SARC/J was used in conjunction with the ZORA basis sets, and the RIJCOSX approximation was applied to decrease computational expense. The conductor-like polarizable continuum model with an input epsilon value of 6.97 (the dielectric constant of 2-methyltetrahedrofuran) was used in all calculations, and dispersion effects were accounted for with the D3BJ keyword.

CASSCF/NEVPT2 calculations based on prior work by Singh et al. were also used in attempt to better model the EPR parameters for $[\mathbf{1}]^{-T}$.[36] The same CPCM model and dispersion effects as described in the DFT section were also present in the CASSCF/NEVPT2 calculations. The input orbitals for the active space were selected from QRO orbitals obtained via the previously-described DFT EPR calculations. In analogy to Singh's methods, the minimal five d orbital basis for the three-coordinate nickel site was initially chosen for the calculation, giving a CASSCF calculation performed with 9 electrons in 5 orbitals (CASSCF(9,5)). This led to overestimation of the largest g-shift (g_{max} (calc'd) = 2.862 compared to g_{max} (exp) = 2.538), likely because the minimal basis does not account for metal-ligand covalency. These results are consistent with Singh's report of large deviations between experimental and calculated g_{max} in $S = 1/2$ d^9 systems including nickel(I) complexes. However, when a larger active space that incorporated a Ni–C σ, two Ni–S σ, and one Ni–Ni σ bonding interaction was used, the calculated g values did not improve, and actually deviated more from experiment (g = [1.988, 1.980, 1.807]). A closer inspection of the output shows that this large deviation is probably because the expanded active space caused the spin density to be localized to the square planar four-coordinate nickel site, as inclusion of the Ni–S and Ni–Ni bonding interactions introduced the four-coordinate nickel site to the active space.

Although DFT calculations successfully reproduced the trend in *g*-values of $g_1 > g_2, g_3$ in both the T- and Y-shaped conformers, the magnitude of g_1 was consistently underestimated (Table S3), CASSCF calculations better reproduced this trend in *g*-values of [**1**]$^-$ (where $g_z >> g_x, g_y$), but significantly overestimated their magnitude. This difficulty in reproducing the magnetic properties of [**1**]$^-$ is likely due to a combination of large spin-orbit coupling, which is caused by the nearly degenerate d_{xy} and d_{x2-y2} orbitals (the latter of which is the SOMO based on crystal field splitting and our calculations), and the presence of soft sulfur-based ligands, which leads to an overestimation of *g*-values in CASSCF calculations due to difficulty in reproducing the covalency of Ni–S bonds.[36]

10.4. DFT Coordinates

[**1**]$^{-}$

Ni	-0.29969700	0.32951000	-0.63122700
Ni	1.91280300	-0.87764500	-1.21191500
S	1.20615300	0.91366300	-2.30876200
S	-0.03105100	-1.89574100	-1.36590900
N	3.54996400	-0.02425000	-0.86469200
N	2.57070100	-2.29539300	-0.17268600
O	4.78486200	1.93203300	-0.87890000
N	-1.80269800	1.53424800	1.55181000
N	-3.02745000	0.02123400	0.64106200
O	2.14111500	-4.32164000	0.86449100
C	4.45507200	-0.85812600	-0.18421100
C	3.91682200	-2.12170900	0.18345700
C	2.60008700	2.03838000	-1.81199600
C	-1.77332900	0.58795300	0.56516300
C	4.72202400	-3.04150300	0.86265400
H	4.30198300	-3.99519500	1.13516600
C	5.78023600	-0.55516000	0.14316300
H	6.17557400	0.40763100	-0.13363300
C	6.56589200	-1.48585800	0.81915300
H	7.59340800	-1.23716000	1.06091600
C	3.76889100	1.27530100	-1.13630500
C	-3.52981000	-1.00332000	-0.23513300
C	0.32727600	2.28824700	2.51335500
C	-1.00918800	3.78136600	1.08676600
C	-0.79964400	2.54803800	1.72535000
C	-3.62043900	-0.73121300	-1.61150500
C	6.04029900	-2.71995200	1.17674400
H	6.65303700	-3.44538500	1.70063600
C	0.27366900	-3.10047800	0.01155700
C	2.09669400	3.08790500	-0.82141100
H	1.33979500	3.72093600	-1.28470000
H	2.93076900	3.70960500	-0.48925800
H	1.65083600	2.60971400	0.04864600
C	1.79024500	-3.31336600	0.24283000
C	-3.79433000	0.62747000	1.62972200

C	-3.02350200	1.56843800	2.20784100
C	-0.08276600	4.79432300	1.31975300
H	-0.20344100	5.75208000	0.83052000
C	-2.14736200	3.99166400	0.09904000
H	-2.84681700	3.16247700	0.19929900
C	-3.94317300	-2.23717500	0.30434000
C	-0.41185600	-4.42099600	-0.33473900
H	-1.48634900	-4.27022900	-0.45768300
H	-0.23000300	-5.14930200	0.45754300
H	-0.01721900	-4.82654900	-1.26709000
C	0.63375600	0.91230500	3.07493000
H	-0.19262600	0.25167300	2.81370100
C	3.12870500	2.71741000	-3.08004200
H	3.46896100	1.97664900	-3.80494600
H	3.97090300	3.36000700	-2.81763200
H	2.34426800	3.31876500	-3.54578400
C	-3.32767600	0.63570200	-2.21146400
H	-2.75962600	1.21289800	-1.48801600
C	-2.46134900	0.56028100	-3.47252000
H	-1.55458200	-0.01203300	-3.28246400
H	-2.16051500	1.56443000	-3.77567900
H	-2.99546000	0.10449700	-4.30969000
C	-0.26458800	-2.53065500	1.32916400
H	0.25534900	-1.60725500	1.56841700
H	-0.10971400	-3.24898100	2.13868200
H	-1.32266400	-2.29958500	1.24162800
C	1.01477600	4.58085300	2.14044200
H	1.73289500	5.37571600	2.29903700
C	1.22322600	3.33896300	2.71619600
H	2.11340700	3.16835000	3.30678200
C	-4.39777900	-3.21350100	-0.58082500
H	-4.70556700	-4.17581500	-0.19506100
C	-4.07503500	-1.74862000	-2.44731000
H	-4.13407100	-1.56860800	-3.51197900
C	-3.97828600	-2.53490100	1.79905500
H	-3.34529300	-1.80832500	2.30900400
C	-1.61351200	3.95491900	-1.34141500
H	-0.92887700	4.78628200	-1.52426800

H	-2.43929800	4.03081600	-2.05326000
H	-1.07257400	3.02786800	-1.53902800
C	-4.44860700	-2.98273400	-1.94463700
H	-4.78390000	-3.76444900	-2.61506800
C	-3.45191000	-3.93175800	2.16258800
H	-3.36973900	-4.02178400	3.24799000
H	-2.47279100	-4.12324000	1.73284800
H	-4.13050900	-4.71679200	1.82278500
C	-2.93924700	5.27601000	0.36993500
H	-3.32197200	5.29714500	1.39257000
H	-3.78800500	5.34510800	-0.31434700
H	-2.32638000	6.16762100	0.22301800
C	-4.64169000	1.38762900	-2.47401100
H	-5.25637400	0.85919400	-3.20724300
H	-4.43386700	2.38761600	-2.86281300
H	-5.22792700	1.49306900	-1.55810900
C	1.90166000	0.34405500	2.41878100
H	1.79165100	0.27702500	1.33540200
H	2.11094300	-0.66087000	2.78785600
H	2.77432500	0.96620600	2.62514700
C	-5.41267400	-2.38624800	2.33944200
H	-6.06221200	-3.14264200	1.89296600
H	-5.84676800	-1.41327900	2.10852000
H	-5.43112100	-2.52039700	3.42376600
C	0.74737000	0.92560900	4.60484400
H	1.58466500	1.54291500	4.93786600
H	0.91682400	-0.08771200	4.97474500
H	-0.16259300	1.31317700	5.06964000
H	-3.22705100	2.26283800	3.00243400
H	-4.80738800	0.33701100	1.82733100

[**1**]$^{-}$ Y-geometry

Ni	0.22932	-0.00006	-0.06283
Ni	-2.28150	0.00010	-0.55070
S	-0.89427	-1.69146	-0.97013
S	-0.89410	1.69154	-0.96999
N	-3.63610	-1.28044	-0.24143
N	-3.63598	1.28073	-0.24136

O	-4.23041	-3.53505	-0.06601
N	2.96779	-1.08722	-0.12983
N	2.96794	1.08680	-0.13011
O	-4.23011	3.53537	-0.06578
C	-4.92537	-0.71681	-0.16528
C	-4.92531	0.71720	-0.16523
C	-1.88304	-2.97715	-0.02000
C	2.08926	-0.00013	-0.00813
C	-6.15310	1.41664	-0.07981
H	-6.12412	2.51277	-0.07913
C	-6.15323	-1.41614	-0.07989
H	-6.12435	-2.51228	-0.07928
C	-7.36295	-0.70276	-0.00056
H	-8.31435	-1.25879	0.05896
C	-3.38790	-2.62195	-0.14391
C	2.62286	2.48597	-0.08063
C	2.51749	-3.12623	1.18646
C	2.51645	-3.20378	-1.30115
C	2.62254	-2.48632	-0.07979
C	2.51631	3.20282	-1.30230
C	-7.36289	0.70336	-0.00052
H	-8.31424	1.25947	0.05903
C	-1.88277	2.97731	-0.01984
C	-1.49785	-2.93622	1.46817
H	-0.41834	-3.16049	1.58820
H	-2.09258	-3.68442	2.03793
H	-1.70215	-1.92917	1.88443
C	-3.38767	2.62222	-0.14374
C	4.28767	0.68299	-0.34389
C	4.28758	-0.68365	-0.34371
C	2.32687	-4.59988	-1.22796
H	2.23587	-5.18020	-2.15980
C	2.62680	-2.51493	-2.66299
H	2.58410	-1.42092	-2.48016
C	2.51842	3.12653	1.18534
C	-1.61215	4.35747	-0.62715
H	-0.55944	4.66489	-0.46273
H	-2.29719	5.09339	-0.15613

H	-1.81621	4.35834	-1.71725
C	2.64947	-2.33200	2.48904
H	2.21650	-1.32859	2.28347
C	-1.61249	-4.35732	-0.62733
H	-1.81656	-4.35817	-1.71742
H	-2.29757	-5.09322	-0.15632
H	-0.55981	-4.66482	-0.46292
C	2.62618	2.51326	-2.66383
H	2.58288	1.41937	-2.48045
C	1.44975	2.85936	-3.59888
H	0.48087	2.60274	-3.12219
H	1.53580	2.28614	-4.54655
H	1.43696	3.93802	-3.86690
C	-1.49758	2.93635	1.46834
H	-1.70192	1.92930	1.88458
H	-2.09230	3.68455	2.03810
H	-0.41807	3.16059	1.58840
C	2.23631	-5.25506	0.00646
H	2.08317	-6.34581	0.04181
C	2.32046	-4.52161	1.20022
H	2.22997	-5.04604	2.16337
C	2.32144	4.52193	1.19849
H	2.23140	5.04683	2.16143
C	2.32685	4.59896	-1.22973
H	2.23550	5.17882	-2.16182
C	2.65120	2.33305	2.48828
H	2.21901	1.32918	2.28335
C	1.45021	-2.86085	-3.59793
H	1.43673	-3.93968	-3.86520
H	1.53674	-2.28833	-4.54597
H	0.48146	-2.60329	-3.12150
C	2.23681	5.25477	0.00440
H	2.08374	6.34555	0.03927
C	1.86385	2.93825	3.66446
H	1.87835	2.24134	4.52771
H	0.80424	3.12374	3.39791
H	2.30416	3.89764	4.01286
C	3.98152	-2.83029	-3.33671

H	4.84137	-2.53516	-2.70003
H	4.07332	-2.28903	-4.30257
H	4.08022	-3.91688	-3.54992
C	3.98103	2.82758	-3.33778
H	4.08037	3.91404	-3.55135
H	4.07241	2.28595	-4.30347
H	4.84077	2.53212	-2.70110
C	1.86244	-2.93722	3.66542
H	0.80301	-3.12368	3.39885
H	1.87631	-2.23986	4.52830
H	2.30346	-3.89610	4.01435
C	4.13440	2.15105	2.88519
H	4.61214	3.13657	3.07640
H	4.71759	1.63653	2.09569
H	4.21879	1.54677	3.81364
C	4.13244	-2.14856	2.88613
H	4.61100	-3.13358	3.07785
H	4.21613	-1.54382	3.81435
H	4.71531	-1.63384	2.09652
H	5.10411	-1.40146	-0.46980
H	5.10430	1.40065	-0.47017

[**2**]$^{-}$

Ni	-0.19162000	-0.00001900	0.35388200
Ni	2.38284700	0.00009100	-0.62487700
S	0.95579200	1.60730800	-1.09154300
S	0.95570300	-1.60707200	-1.09141200
N	3.65458700	1.28401600	-0.12422500
N	3.65456700	-1.28388700	-0.12430700
O	4.14605800	3.51228100	0.26331700
N	-3.00415100	1.06849200	-0.08460800
N	-3.00409000	-1.06860400	-0.08457600
O	4.14591300	-3.51211600	0.26359500
C	4.90182000	0.71092500	0.17664300
C	4.90180700	-0.71082300	0.17662300
C	1.87671900	2.97124500	-0.22949800
C	-2.16047500	-0.00004000	-0.01679200
C	6.08506600	-1.39840800	0.46337900

H	6.07220800	-2.47532100	0.46267900
C	6.08510200	1.39847200	0.46341400
H	6.07227000	2.47538600	0.46273100
C	7.25339100	0.69415400	0.74362700
H	8.16425600	1.24182800	0.95912300
C	3.36845600	2.59767100	-0.02745300
C	-2.62425200	-2.45581000	-0.07742000
C	-2.61689500	3.13329000	1.14615400
C	-2.33213000	3.08101300	-1.29921800
C	-2.62435300	2.45569000	-0.07715000
C	-2.33195000	-3.08083900	-1.29962400
C	7.25337400	-0.69412700	0.74361000
H	8.16422600	-1.24183100	0.95909500
C	1.87667100	-2.97110700	-0.22959200
C	1.28886300	3.22803300	1.16117000
H	0.24134200	3.51678000	1.08326800
H	1.84903600	4.02773600	1.65215800
H	1.35458400	2.32784900	1.77115500
C	3.36839100	-2.59751700	-0.02744300
C	-4.33276200	-0.67335100	-0.15162000
C	-4.33279900	0.67316000	-0.15159000
C	-2.08364800	4.45084200	-1.26667900
H	-1.83721700	4.97027100	-2.18150900
C	-2.28388100	2.29590500	-2.60217500
H	-1.90484900	1.30275100	-2.35581900
C	-2.61666100	-3.13364400	1.14575700
C	1.77545500	-4.23437700	-1.08779800
H	0.73408000	-4.54669900	-1.18136300
H	2.35392400	-5.03417400	-0.62301600
H	2.17686400	-4.05756600	-2.08670800
C	-2.85911100	2.41164200	2.46123500
H	-2.71015700	1.34664900	2.28301400
C	1.77542600	4.23466900	-1.08747000
H	2.17683500	4.05808600	-2.08642200
H	2.35386900	5.03441000	-0.62256100
H	0.73403800	4.54696100	-1.18095200
C	-2.28372800	-2.29544100	-2.60241100
H	-1.90472600	-1.30232700	-2.35581900

C	-1.32320400	-2.89088500	-3.63554700
H	-0.33444800	-3.04375800	-3.20731600
H	-1.22079700	-2.20010400	-4.47476000
H	-1.69442500	-3.83777100	-4.03677400
C	1.28878200	-3.22813700	1.16101500
H	1.35451000	-2.32807100	1.77117600
H	1.84892500	-4.02793900	1.65187800
H	0.24125700	-3.51684000	1.08304300
C	-2.11498600	5.15838600	-0.07299000
H	-1.90786000	6.22141400	-0.07314200
C	-2.36945300	4.50571000	1.12147600
H	-2.35126500	5.06233400	2.04871700
C	-2.36907500	-4.50603600	1.12079800
H	-2.35073100	-5.06283700	2.04792500
C	-2.08335300	-4.45064900	-1.26736100
H	-1.83689900	-4.96988600	-2.18229400
C	-2.85884900	-2.41220800	2.46096500
H	-2.70957100	-1.34723300	2.28294400
C	-1.32339800	2.89161800	-3.63519700
H	-1.69466900	3.83858900	-4.03617700
H	-1.22098400	2.20104300	-4.47458200
H	-0.33464200	3.04442900	-3.20695800
C	-2.11459200	-5.15843600	-0.07381200
H	-1.90736000	-6.22144400	-0.07419400
C	-1.85934800	-2.82577900	3.54788500
H	-1.96811000	-2.17695000	4.41862300
H	-0.83420500	-2.74049600	3.19207800
H	-2.02372700	-3.85399000	3.87747700
C	-3.68448500	2.14147900	-3.21933500
H	-4.37738500	1.63732100	-2.54737900
H	-3.62740400	1.55415400	-4.13882200
H	-4.10202900	3.12070000	-3.46842700
C	-3.68432700	-2.14091500	-3.21953400
H	-4.10188900	-3.12008700	-3.46878300
H	-3.62724100	-1.55345400	-4.13893700
H	-4.37721300	-1.63684900	-2.54749700
C	-1.85933200	2.82477700	3.54807700
H	-0.83428200	2.73935800	3.19205400

H	-1.96806600	2.17581500	4.41872100
H	-2.02343900	3.85296700	3.87786000
C	-4.30386800	-2.60930200	2.94352600
H	-4.50810200	-3.66758200	3.12462900
H	-5.02331500	-2.24872700	2.20671700
H	-4.47388000	-2.06607200	3.87623900
C	-4.30403500	2.60900700	2.94395000
H	-4.50804000	3.66729700	3.12523600
H	-4.47412400	2.06566600	3.87658500
H	-5.02358700	2.24872000	2.20709000
C	-0.10389000	-0.00023100	2.16154100
O	-0.09561100	-0.00065500	3.30814700
H	-5.13706600	1.38327700	-0.20103700
H	-5.13697800	-1.38352000	-0.20111000

TSc (TS5)

Ni	-0.20452500	0.11367400	-0.34623500
Ni	2.03208000	-0.55496200	-1.04052200
S	1.02753000	1.17163300	-1.99496800
S	0.31430100	-1.86705600	-1.39734000
N	3.49340100	0.53065000	-0.58382400
N	2.93917300	-1.95214600	-0.17909000
O	4.43595900	2.66390500	-0.48958500
N	-2.25848800	1.64263000	1.07488700
N	-3.03133700	-0.30146100	0.45768700
O	2.92654400	-4.15792900	0.58868300
C	4.48874500	-0.16593900	0.13732900
C	4.17813000	-1.54840600	0.36502400
C	2.37972200	2.44340700	-1.70283800
C	-1.86196800	0.47406600	0.41623500
C	5.09556800	-2.36266400	1.07168100
H	4.82918000	-3.41267400	1.24047000
C	5.70983500	0.36372100	0.61959800
H	5.92010600	1.42444600	0.43890500
C	6.60986600	-0.46374700	1.31462200
H	7.55798500	-0.03804700	1.68550000
C	3.53992100	1.87307600	-0.84073600
C	-3.23830000	-1.61228700	-0.11474600

C	-0.57509500	3.10851800	2.16589600
C	-1.90397300	3.92245500	0.23289700
C	-1.53346200	2.89039400	1.14072100
C	-3.19610000	-1.77482500	-1.53203000
C	6.30440500	-1.81744500	1.53967100
H	7.01040400	-2.46318800	2.08945800
C	1.07761100	-3.46862900	-0.77767500
C	1.80686800	3.69349600	-1.02505100
H	1.07775700	4.20643300	-1.68600400
H	2.64138300	4.38931800	-0.79374400
H	1.29318300	3.43571700	-0.07879600
C	2.41288700	-3.20707900	-0.02940000
C	-4.07558600	0.37240400	1.10103400
C	-3.59018800	1.58533100	1.49561700
C	-1.33148600	5.19846400	0.41011200
H	-1.60322800	6.01111200	-0.28299300
C	-2.85518200	3.68272800	-0.94096200
H	-3.20393100	2.63223300	-0.88357500
C	-3.57735000	-2.69198700	0.74958000
C	1.40210000	-4.32230800	-2.02229000
H	0.47723600	-4.56313600	-2.58760200
H	1.88780700	-5.26927200	-1.70088600
H	2.09553700	-3.78257700	-2.69880800
C	-0.04735200	1.97448100	3.03809700
H	-0.75737200	1.12454200	2.94798300
C	2.96174400	2.80097400	-3.08633900
H	3.36532200	1.89960800	-3.59099600
H	3.78878600	3.53175500	-2.95458400
H	2.18181700	3.24690900	-3.73927900
C	-2.87504900	-0.60549500	-2.46232100
H	-2.05819900	-0.03659400	-1.95771700
C	-2.32157100	-1.02722000	-3.83232500
H	-1.44718800	-1.69889000	-3.71152300
H	-1.97564300	-0.12865000	-4.38385000
H	-3.08487200	-1.53034100	-4.46681800
C	0.09522300	-4.21018000	0.13395900
H	-0.21968100	-3.56799500	0.97969000
H	0.59794700	-5.11128700	0.54537300

H	-0.81684300	-4.51123900	-0.42183000
C	-0.41584700	5.44588700	1.44117700
H	0.02027100	6.45029800	1.56498300
C	-0.03014500	4.40359100	2.29405600
H	0.72146600	4.59342300	3.07605700
C	-3.89676100	-3.93548500	0.16248000
H	-4.16253800	-4.78338600	0.81358700
C	-3.50422500	-3.04522600	-2.05653300
H	-3.46450500	-3.19666200	-3.14518600
C	-3.61571200	-2.56725000	2.27684600
H	-3.13991600	-1.60196500	2.54561800
C	-2.11094700	3.83339300	-2.28537900
H	-1.73189400	4.86838700	-2.42837000
H	-2.79228500	3.60676700	-3.13342500
H	-1.24650500	3.13956000	-2.33855300
C	-3.86223600	-4.11551100	-1.22456700
H	-4.10483700	-5.09788100	-1.66157400
C	-2.80420100	-3.67776200	2.97577000
H	-2.75322000	-3.48611500	4.06856400
H	-1.76821500	-3.72414000	2.58845000
H	-3.26550600	-4.67925700	2.83801500
C	-4.10254800	4.58819000	-0.87823400
H	-4.66070100	4.45236000	0.07183000
H	-4.79476300	4.35578300	-1.71537700
H	-3.83387700	5.66358900	-0.95850100
C	-4.10288200	0.31688200	-2.62717300
H	-4.93538400	-0.22062600	-3.13261000
H	-3.84446600	1.20097100	-3.24770400
H	-4.48580200	0.68650700	-1.65360700
C	1.30685700	1.50777800	2.46031800
H	1.16268400	1.10936100	1.42800800
H	1.75663800	0.70288500	3.07480500
H	2.03506900	2.34364200	2.40342700
C	-5.06490500	-2.56354100	2.81475100
H	-5.57618800	-3.52400200	2.58756300
H	-5.67961400	-1.75300700	2.37267500
H	-5.07467100	-2.43061300	3.91793100
C	0.05334800	2.34260200	4.53051800

H	0.81556400	3.12882300	4.71800800
H	0.35755900	1.45331500	5.12129800
H	-0.91599100	2.70659700	4.93291400
H	-4.06753400	2.41477500	2.02635300
H	-5.07089700	-0.06766800	1.20828000
C	-0.05264500	-1.58831000	2.67672200
O	1.01747300	-1.80073000	3.02965400

11.0. References

(1) Drennan, C. *Life on CO_2: Structural Elucidation of the Wood-Ljungdahl Pathway*. Drennan Lab MIT. https://drennan.mit.edu/index.php/research/research-interests/life-on-co2-structural-elucidation-of-the-wood-ljungdahl-pathway/ (accessed 2023-12-13).

(2) Hanss, J.; Krüger, H.-J. First Isolation and Structural Characterization of a Nickel(III) Complex Containing Aliphatic Thiolate Donors. *Angew. Chem. Int. Ed.* **1998**, *37* (3), 360–363.

(3) Dible, B. R.; Sigman, M. S.; Arif, A. M. Oxygen-Induced Ligand Dehydrogenation of a Planar Bis-μ-Chloronickel(I) Dimer Featuring an NHC Ligand. *Inorg. Chem.* **2005**, *44* (11), 3774–3776.

(4) Dolomanov, O. V.; Bourhis, L. J.; Gildea, R. J.; Howard, J. A. K.; Puschmann, H. OLEX2: A Complete Structure Solution, Refinement and Analysis Program. *J. Appl. Crystallogr.* **2009**, *42*, 339–341.

(5) Sheldrick, G. M. Crystal Structure Refinement with It SHELXL. *Acta Crystallogr. Sect. A* **2015**, *71* (1), 3–8.

(6) Stavropoulos, P.; Muetterties, M. C.; Carrie, M.; Holm, R. H. Structural and Reaction Chemistry of Nickel Complexes in Relation to Carbon Monoxide Dehydrogenase: A Reaction System Simulating Acetyl-Coenzyme A Synthase Activity. *J. Am. Chem. Soc.* **1991**, *113* (22), 8485–8492.

(7) Mandimutsira, B. S.; Yamarik, J. L.; Brunold, T. C.; Gu, W.; Cramer, S. P.; Riordan, C. G. Dioxygen Activation by a Nickel Thioether Complex: Characterization of a $Ni^{III}_2(\mu\text{-}O)_2$ Core. *J. Am. Chem. Soc.* **2001**, *123* (37), 9194–9195.

(8) Fujita, K.; Rheingold, A. L.; Riordan, C. G. Thioether-Ligated Nickel(I) Complexes for the Activation of Dioxygen. *Dalton Trans.* **2003**, 10, 2004–2008.

(9) Yoo, C.; Lee, Y. A T-Shaped Nickel(I) Metalloradical Species. *Angew. Chem. Int. Ed.* **2017**, *56* (32), 9502–9506.

(10) Stojanovic, R. S.; Bond, A. M. Examination of Conditions under Which the Reduction of the Cobaltocenium Cation Can Be Used as a Standard Voltammetric Reference Process in Organic and Aqueous Solvents. *Anal. Chem.* **1993**, *65*, 56–64.

(11) Reineke, M. H.; Porter, T. M.; Ostericher, A. L.; Kubiak, C. P. Synthesis and Characterization of Heteroleptic Ni(II) Bipyridine Complexes Bearing Bis(N-Heterocyclic Carbene) Ligands. *Organometallics* **2018**, *37* (3), 448–453.

(12) Tian, Y. M.; Guo, X. N.; Kuntze-Fechner, M. W.; Krummenacher, I.; Braunschweig, H.; Radius, U.; Steffen, A.; Marder, T. B. Selective Photocatalytic C-F Borylation of Polyfluoroarenes by Rh/Ni Dual Catalysis Providing Valuable Fluorinated Arylboronate Esters. *J. Am. Chem. Soc.* **2018**, *140* (50), 17612–17623.

(13) Luca, O. R.; Thompson, B. A.; Takase, M. K.; Crabtree, R. H. Synthesis and Electrochemistry of a Series of Cyclopentadienyl Ni N-Heterocyclic Carbene Compounds. *J. Organomet. Chem.* **2013**, *730*, 79–83.

(14) Diccianni, J. B.; Katigbak, J.; Hu, C.; Diao, T. Mechanistic Characterization of (Xantphos)Ni(I)-Mediated Alkyl Bromide Activation: Oxidative Addition, Electron Transfer, or Halogen-Atom Abstraction. *J. Am. Chem. Soc.* **2019**, *141* (4), 1788–1796.

(15) Kitiachvili, K. D.; Mindiola, D. J.; Hillhouse, G. L. Preparation of Stable Alkyl Complexes of Ni(I) and Their One-Electron Oxidation to Ni(II) Complex Cations. *J. Am. Chem. Soc.* **2004**, *126* (34), 10554–10555.

(16) Hu, X.; Castro-Rodriguez, I.; Meyer, K. Synthesis and Characterization of Electron-Rich Nickel Tris-Carbene Complexes. *Chem. Commun.* **2004**, No. 19, 2164–2165.

(17) Mindiola, D. J.; Hillhouse, G. L. Terminal Amido and Imido Complexes of Three-Coordinate Nickel. *J. Am. Chem. Soc.* **2001**, *123* (19), 4623–4624.

(18) Laskowski, C. A.; Hillhouse, G. L. Two-Coordinate d^9 Complexes. Synthesis and Oxidation of NHC Nickel(I) Amides. *J. Am. Chem. Soc.* **2008**, *130* (42), 13846–13847.

(19) Krzystek, J.; Sienkiewicz, A.; Pardi, L.; Brunel, L. DPPH as a Standard for High-Field EPR. *J. Magn. Reson.* **1997**, *125* (1), 207–211.

(20) Stoll, S.; Schweiger, A. EasySpin, a Comprehensive Software Package for Spectral Simulation and Analysis in EPR. *J. Magn. Reson.* **2006**, *178* (1), 42–55.
(21) van der Est, A.; Goldfarb, D.; Stoll, S. Continuous-Wave EPR. *EPR Spectroscopy: Fundamentals and Methods* **2018**, 8–9.
(22) Pietrzyk, P.; Podolska, K.; Sojka, Z. Resolving Conformation Dichotomy for Y- and T-Shaped Three-Coordinate Ni^{I} Carbonyl Complexes with Relativistic DFT Analysis of EPR Fingerprints. *Chem. Eur. J.* **2009**, *15* (44), 11802–11807.
(23) Kisgeropoulos, E. C.; Manesis, A. C.; Shafaat, H. S. Ligand Field Inversion as a Mechanism to Gate Bioorganometallic Reactivity: Investigating a Biochemical Model of Acetyl CoA Synthase Using Spectroscopy and Computation. *J. Am. Chem. Soc.* **2021**, *143* (2), 849–867.
(24) Can, M.; Giles, L. J.; Ragsdale, S. W.; Sarangi, R. X-Ray Absorption Spectroscopy Reveals an Organometallic Ni–C Bond in the CO-Treated Form of Acetyl-CoA Synthase. *Biochemistry* **2017**, *56* (9), 1248–1260.
(25) George, S. J.; Seravalli, J.; Ragsdale, S. W. EPR and Infrared Spectroscopic Evidence That a Kinetically Competent Paramagnetic Intermediate Is Formed When Acetyl-Coenzyme A Synthase Reacts with CO. *J. Am. Chem. Soc.* **2005**, *127* (39), 13500–13501.
(26) Bender, G.; Stich, T. A.; Yan, L.; Britt, R. D.; Cramer, S. P.; Ragsdale, S. W. Infrared and EPR Spectroscopic Characterization of a Ni(I) Species Formed by Photolysis of a Catalytically Competent Ni(I)-CO Intermediate in the Acetyl-CoA Synthase Reaction. *Biochemistry* **2010**, *49* (35), 7516–7523.
(27) Stephens, P. J.; Devlin, F. J.; Chabalowski, C. F.; Frisch, M. J. Ab Initio Calculation of Vibrational Absorption and Circular Dichroism Spectra Using Density Functional Force Fields. *J. Phys. Chem.* **1994**, *98* (45), 11623–11627.
(28) Grimme, S. Density Functional Theory with London Dispersion Corrections. *Wiley Interdisciplinary Reviews: Computational Molecular Science* **2011**, *1* (2), 211–228.
(29) Weigend, F. Accurate Coulomb-Fitting Basis Sets for H to Rn. *Phys. Chem. Chem. Phys.* **2006**, *8* (9), 1057–1065.
(30) Thomas, C. M. Metal-Metal Multiple Bonds in Early/Late Heterobimetallic Complexes: Applications Toward Small Molecule Activation And Catalysis. *Comments on Inorganic Chemistry* **2011**, *32* (1), 14–38.
(31) Wilson, D. W. N.; Fataftah, M. S.; Mathe, Z.; Mercado, B. Q.; DeBeer, S.; Holland, P. L. Three-Coordinate Nickel and Metal–Metal Interactions in a Heterometallic Iron–Sulfur Cluster. *J. Am. Chem. Soc.* **2024**, *146* (6), 4013–4025.
(32) Lukoyanov, D. A.; Yang, Z.-Y.; Pérez-González, A.; Raugei, S.; Dean, D. R.; Seefeldt, L. C.; Hoffman, B. M. 13C ENDOR Characterization of the Central Carbon within the Nitrogenase Catalytic Cofactor Indicates That the CFe_6 Core Is a Stabilizing "Heart of Steel." *J. Am. Chem. Soc.* **2022**, *144* (40), 18315–18328.
(33) Paw, W.; Cummings, S. D.; Adnan Mansour, M.; Connick, W. B.; Geiger, D. K.; Eisenberg, R. Luminescent Platinum Complexes: Tuning and Using the Excited State. *Coord. Chem. Rev.* **1998**, *171*, 125–150.
(34) Software Update: The ORCA Program System—Version 5.0. *Wiley Interdiscip. Rev. Comput. Mol. Sci.* **2022**, *12*, e1606.
(35) Podolska-Serafin, K.; Pietrzyk, P. Molecular Structures of Nickel Adducts in Zeolites – Interpretation of Experimental EPR g-Tensors Guided by DFT Calculations. *J. Mol. Struct.* **2019**, *1180*, 754–763.
(36) Singh, S. K.; Atanasov, M.; Neese, F. Challenges in Multireference Perturbation Theory for the Calculations of the G-Tensor of First-Row Transition-Metal Complexes. *J. Chem. Theory Comput.* **2018**, *14* (9), 4662–4677.
